# Tonic type I interferon signaling optimizes the antiviral function of plasmacytoid dendritic cells

**DOI:** 10.1038/s41590-025-02279-4

**Published:** 2025-10-14

**Authors:** Joseph N. Pucella, Raul A. Maqueda-Alfaro, Hai Ni, Fernando Bandeira Sulczewski, Anna Eichinger, Eduardo Esteva, Ai C. Ra, Annesa Das, Oriana A. Perez, Jue Feng, Marlon Stoeckius, Peter Smibert, Alireza Khodadadi-Jamayran, Igor Dolgalev, Ellie Ivanova, Stela Sota, Ken Cadwell, Sergei B. Koralov, Judy Zhong, Chetna Soni, Daniel B. Stetson, Stuart P. Weisberg, Donna L. Farber, Juliana Idoyaga, Boris Reizis

**Affiliations:** 1 Department of Pathology, New York University Grossman School of Medicine, New York, NY USA; 2 Inflammatory Bowel Disease Center, Division of Gastroenterology, Department of Medicine, New York University Grossman School of Medicine, New York, NY USA; 3 Department of Pharmacology, University of California San Diego School of Medicine, San Diego, CA USA; 4 Department of Pediatrics, Dr. von Hauner Children’s Hospital, University Hospital, Ludwig-Maximilians-Universität München, Munich, Germany; 5 Applied Bioinformatics Laboratories, New York University Grossman School of Medicine, New York, NY USA; 6 Technology Innovation Lab, New York Genome Center, New York, NY USA; 7 Department of Microbiology, New York University Grossman School of Medicine, New York, NY USA; 8 Division of Gastroenterology, Department of Medicine, University of Pennsylvania Perelman School of Medicine, Philadelphia, PA USA; 9 Division of Biostatistics, Department of Population Health Sciences, Weill Cornell Medicine, New York, NY USA; 10 Department of Immunology, University of Washington School of Medicine, Seattle, WA USA; 11 Department of Pathology and Cell Biology, Columbia University Irving Medical Center, New York, NY USA; 12 Department of Microbiology and Immunology, Columbia University Irving Medical Center, New York, NY USA; 13 Department of Molecular Biology, University of California San Diego School of Biological Sciences, San Diego, CA USA

**Keywords:** Plasmacytoid dendritic cells, Interferons

## Abstract

Plasmacytoid dendritic cells (pDCs) mount powerful antiviral type I interferon (IFN-I) responses, yet only a fraction of pDCs produces high levels of IFN-I. Here we report that peripheral pDCs in naive mice comprise three subsets (termed A, B and C) that represent progressive differentiation stages. This heterogeneity was generated by tonic IFN-I signaling elicited in part by the cGAS/STING and TLR9 DNA-sensing pathways. A small ‘IFN-I-naive’ subset (pDC-A) could give rise to other subsets; it was expanded in STING deficiency or after the IFN-I receptor blockade, but was abolished by exogenous IFN-I. In response to RNA viruses, pDC-A showed increased Bcl2-dependent survival and superior IFN-I responses, but was susceptible to virus infection. Conversely, the majority of pDCs comprised the ‘IFN-I-primed’ subsets (pDC-B/C) that showed lower IFN-I responses and poor survival, but did not support virus replication. Thus, tonic IFN-I signaling decreases the cytokine-producing capacity and survival of pDCs but increases their virus resistance, facilitating optimal antiviral responses.

## Abstract (web summary)

Pucella, Maqueda-Alfaro and colleagues distinguish three developmentally related subsets of mouse plasmacytoid dendritic cells that differ in their ability to produce type I interferon and their susceptibility to virus.

## Main

IFN-I, comprising interferon-β (IFNβ) and multiple subtypes of interferon-α (IFNα), is the key mediator of antiviral immunity in vertebrates. IFN-I signals through its ubiquitously expressed receptor (IFNAR) to induce hundreds of interferon-stimulated genes (ISGs) that collectively establish an antiviral cellular state^1^. Nonimmune cell types can produce IFN-I once infected by a virus, which is sensed by intracellular nucleic acid sensors such as cGAS/STING for DNA^2^. Intracellular virus sensing leads to the induction of IFNβ, feedback signaling through IFNAR, induction and activation of the key transcription factor (TF) IRF7, and further IRF7-dependent transcription of IFNβ and a few IFNα subtypes.

In addition to this common cell-intrinsic mechanism, IFN-I responses can be initiated by a unique sentinel cell type, the pDC. pDCs sense solution-borne viruses or other virus-infected cells through endosomal Toll-like receptors (TLRs) TLR9 and TLR7 (for DNA and RNA, respectively), and rapidly induce IFNβ and all IFNα genes via IRF7, which is expressed in pDCs in the steady state^3^. Due to their rapid and massive production of IFN-I and other cytokines, pDCs facilitate innate and/or adaptive responses to multiple viruses. The latter include vesicular stomatitis virus (VSV)^4^ and coronaviruses such as murine coronavirus (M-CoV, also known as murine hepatitis virus or MHV)^5,6^. pDCs are short-lived cells that are being continuously generated in the bone marrow (BM) in a process driven by the TF TCF4 (E2-2)^7^, followed by the exit to peripheral lymphoid organs. Cells that are closely related to pDCs^8^, herein referred to as transitional dendritic cells (tDCs)^9^, are also TCF4-dependent yet express distinct markers such as CX3CR1, show weak IFN-I response and instead produce inflammatory cytokines such as IL-1β^10–12^.

Following the activation of human pDCs with viruses or TLR ligands, only a fraction of them produces IFN-I^13–15^. Similarly, <10% of pDCs express IFN-I gene reporters during murine cytomegalovirus infection or when stimulated with CpG in vivo^16,17^, yielding a distinct subset of activated pDCs^18–20^. However, even as some pre-existing phenotypic and/or transcriptional heterogeneity has been reported in murine^21–24^ and human^25–27^ pDCs, neither the purpose nor the mechanism of heterogeneous pDC responses is known. Another open question in pDC biology concerns the apparent resistance of pDCs to multiple viruses including human immunodeficiency virus^28^, cytomegalovirus^14^ or VSV^29^. While such resistance is likely to facilitate IFN-I responses while restricting viral replication, its molecular basis remains to be elucidated.

Here we undertook a comprehensive single-cell profiling of steady-state mouse pDCs, and uncovered transcriptional and phenotypic heterogeneity that was not reflected in the chromatin and was driven by tonic IFN-I signaling. Prospective isolation of pDC subsets revealed a progressive decrease of cytokine-producing capacity along with the acquisition of antiviral resistance in ‘IFN-I-experienced’ pDCs. We propose that the diminished cytokine response in the majority of peripheral pDCs represents a trade-off for their antiviral resistance and hence a more robust response to infection.

## Results

### Single-cell multiomics reveals heterogeneity in steady-state pDCs

**Fig. 1:**
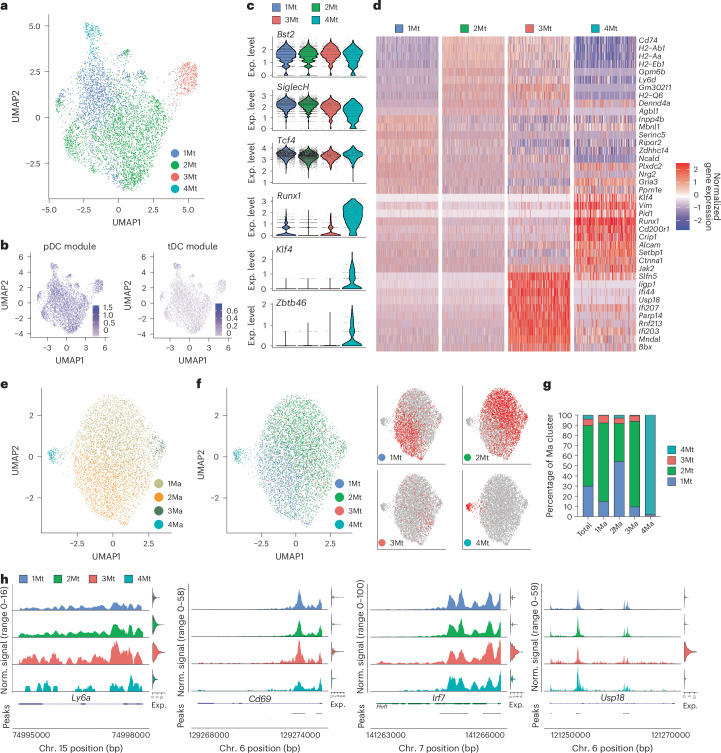
Mature splenic pDCs show transcriptional heterogeneity. Sorted pDCs from pooled spleens of naive mice were subjected to multiome analysis by snRNA-seq and snATAC-seq. Data shown are from one experiment. **a**–**d**, Heterogeneity of pDCs by transcriptome (RNA-seq). **a**, UMAP plot of cells based on messenger RNA transcript reads, indicating Mt clusters. **b**, Feature plots of combined transcriptional scores for pDC or tDC cell types projected onto the UMAP plot from **a**. **c**, The expression of key pDC (*Bst2*, *SiglecH*, *Tcf4*) and tDC (*Runx1*, *Klf4*) transcripts within Mt clusters. **d**, Heatmap of top ten marker genes of each Mt (rows) across individual cells (columns). **e**–**i**, Heterogeneity of pDCs by open chromatin (ATAC-seq). **e**, UMAP plot of cells based on ATAC-seq peaks, showing multiome ATAC-seq (Ma) clusters. **f**, The projection of Mt clusters onto the UMAP plot based on ATAC-seq from **e**. Shown are combined projections of all clusters (left) and feature plots of individual Mt clusters (right). **g**, The fraction of Mt clusters within the total dataset or within each Ma cluster. **h**, Combined ATAC-seq profiles of all cells within each Mt cluster across interferon-inducible genes that distinguish the 3Mt cluster. Shown are normalized ATAC-seq peaks, with the associated transcript expression shown as violin plots on the right. Exp., expression; Norm., normalized. Source data

**Extended Data Fig. 1:**
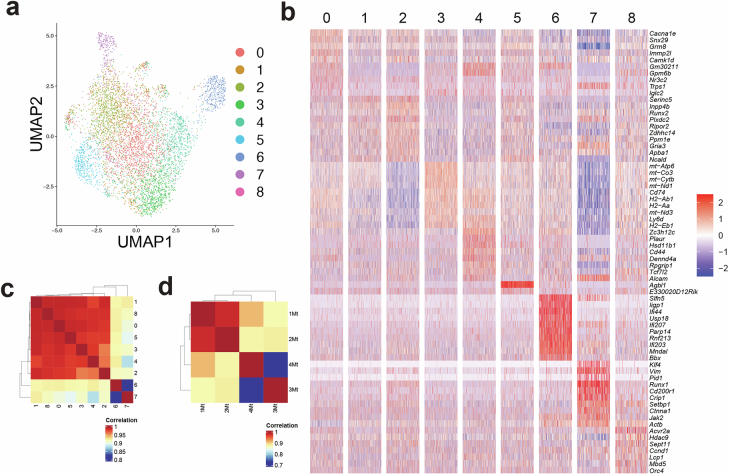
Initial analysis of the single-cell transcriptome of splenic pDCs. Sorted pDCs from pooled spleens of naive mice were subjected to multiome analysis by single-nuclei RNA-seq and ATAC-seq (Fig. 1), and the former was analyzed. (**a**) UMAP plot of cells based on mRNA transcript reads, indicating the original 9 clusters. (**b**) Heatmap of top 10 marker genes of each cluster (rows) across individual cells (columns). (**c**) Correlation heatmap of the original 9 clusters. (**d**) Correlation heatmap of the reduced clusters (Mt clusters in Fig. 1a). Source data

We sorted splenic pDCs from naive C57BL/6 (B6) wild-type (WT) mice and examined them by a multiomic approach combining single-nucleus RNA sequencing (snRNA-seq) and single-nucleus assay for transposase-accessible chromatin with high-throughput sequencing (snATAC-seq). Substantial transcriptional heterogeneity was observed in the resulting 5,828 cells, as depicted by uniform manifold approximation and projection (UMAP) of RNA transcripts (Fig. 1a). The initial nine multiome-derived transcriptome (Mt) clusters (Extended Data Fig. 1a–c) were reduced to four clusters with a lower overlap (Fig. 1a) and reduced marker gene correlation (Extended Data Fig. 1d). The composite pDC module and its constituent pDC-specific genes such as *Bst2*, *Siglech* and *Tcf4* were uniformly high in all clusters (Fig. 1b,c and Supplementary Table 1). In contrast, the expression of a composite tDC module and its constituent genes such as *Klf4*, *Runx1* and *Zbtb46* were detected in a single minor cluster, 4Mt (Fig. 1b,c), which was therefore designated as tDCs and retained for comparison with pDC clusters. Notably, the small cluster 3Mt was distinguished by the expression of ISGs exemplified by *Slfn5*, *Ifi/Ifit* and *Oas/Oasl* genes, *Isg15*, *Usp18, Irf7* and *Cd69* (Fig. 1d and Supplementary Table 1). Clusters 1Mt and 2Mt showed lower expression of MHC class II and several other genes including *Gpm6b* and *Ly6a* in the former (Fig. 1d and Supplementary Table 1).

**Extended Data Fig. 2:**
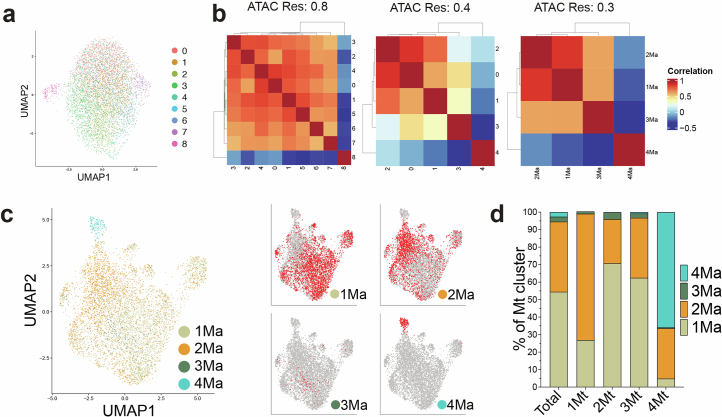
Initial analysis of the single-cell chromatin of splenic pDCs. Sorted pDCs from pooled spleens of naive mice were subjected to multiome analysis by single-nuclei RNA-seq and ATAC-seq (Fig. 1), and the latter was analyzed. (**a**) UMAP plot of cells based on ATAC-Seq reads, indicating the original 9 clusters. (**b**) Correlation heatmap of the original 9 clusters and after cluster reduction, with the corresponding Leiden clustering resolutions indicated. The heatmap on the right corresponds to the four Ma clusters in Fig. 1e. (**c**) The projection of Ma clusters onto the UMAP plot based on RNA-Seq from Fig. 1a. Shown are combined projections of all clusters (left panel) and feature plots of individual Mt clusters (right panel). (**d**) The fraction of Ma clusters within the total dataset and within each Mt cluster. Source data

As in the case of transcriptome, the initial clustering based on ATAC-seq reads was reduced from nine to four multiome-derived ATAC-seq (Ma) clusters with optimal marker gene correlation (Extended Data Fig. 2a,b). However, Ma clusters 1–3 were closely associated on the UMAP projection and had few specific peaks, with only one cluster (4Ma) segregating (Fig. 1e and Supplementary Table 2). The projection of Mt clusters onto the ATAC-seq UMAP revealed a correspondence of the minor tDC cluster 4Mt to cluster 4Ma (Fig. 1f,g), whereas pDC clusters 1Mt–3Mt showed little correlation with the respective Ma clusters (Fig. 1f,g). This was confirmed by a reciprocal projection of Ma clusters onto the transcriptome UMAP (Extended Data Fig. 2c,d). Indeed, ISGs such as *Ly6a*, *Cd69*, *Irf7* and *Usp18*, which were enriched in cluster 3Mt, showed comparable chromatin profiles across all Ma clusters (Fig. 1h). Thus, pDCs exhibit transcriptional heterogeneity that is not mirrored in their chromatin landscape and does not reflect the presence of related cell types such as tDCs.

### pDC heterogeneity is driven by the interferon signature

**Fig. 2:**
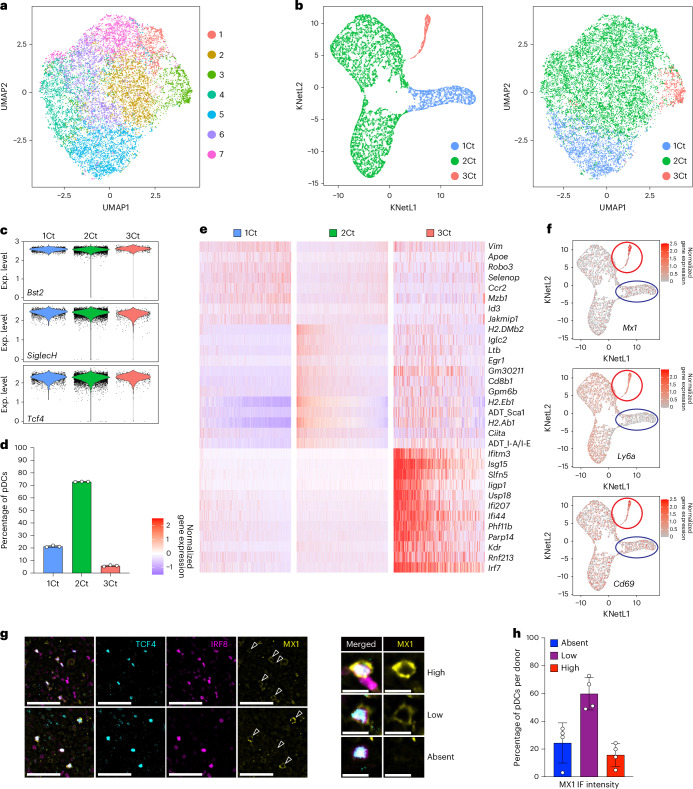
Mature splenic pDCs comprise three transcriptionally and phenotypically distinct subsets. **a**–**f**, Sorted pDCs from spleens of three individual naive mice were hashtagged and analyzed by CITE-seq for single-cell transcriptome and phenotype (ADT). Data shown are from one experiment. **a**, UMAP plot and clustering (clusters are numbered and colored randomly). **b**, Clustering by KNetL. Shown is the KNetL plot with three CITE-seq transcriptome-based (Ct) clusters (left), and the UMAP plot from **a** with the projected Ct clusters derived from KNetL. **c**, Violin plots showing transcript levels of key pDC identity genes by Ct clusters. **d**, Frequencies of Ct clusters within the total sample population. Bars represent means ± s.d.; symbols represent biological replicates (hashtagged samples from individual mice) from one experiment. **e**, Heatmap of marker gene expression based on mRNA transcripts or ADT reads for Ct clusters. **f**, Feature plots of key marker gene transcripts on the KNetL plot from **b**. **g**,**h**, Multiplexed immunofluorescence staining of lymph nodes from healthy human organ donors, stained for pDC markers IRF8 and TCF4, and the ISG MX1. **g**, Single-channel and merged images from two samples; IRF8^+^TCF4^+^ pDCs in the MX1 staining panel are indicated by open arrowheads. Right panels show representative IRF8^+^TCF4^+^ pDCs with high, low or absent MX1 expression. Scale bars, 50 μm (left panels), 10 μm (magnified panels). Representative of four experiments (see **h**). **h**, The frequency of pDCs with different MX1 expression levels. Bars represent means ± s.d.; symbols represent biological replicates (individual donors) from four experiments. IF, immunofluorescence. Source data

**Extended Data Fig. 3:**
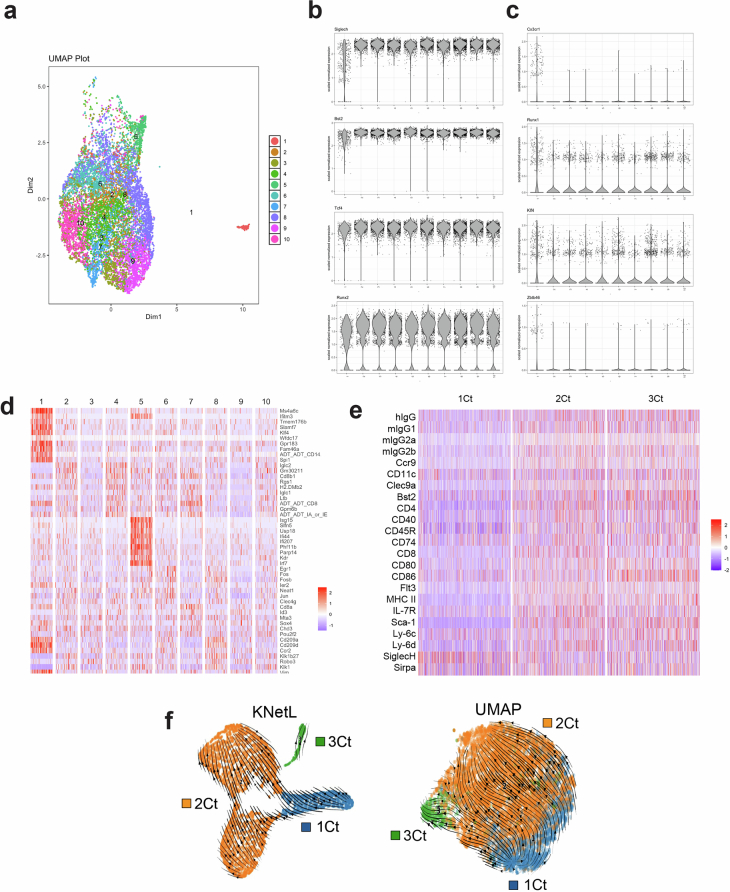
Additional analysis of CITE-Seq of splenic pDCs. Sorted pDCs from spleens of three individual naive mice were hashtagged and analyzed by CITE-Seq for single-cell transcriptome and phenotype (antibody-derived tags, ADT). (**a**) UMAP plot and clustering of the dataset prior to the tDC cluster removal (clusters are numbered and colored randomly). (**b**-**c**) Violin plots showing transcript levels of key pDC (**b**) and tDC (**c**) identity genes. (**d**) Heatmap of top 10 marker gene expression based on mRNA transcripts or ADT reads for the clusters in panel A. (**e**) Heatmap of ADT for markers expressed in pDCs across the three Ct clusters defined in Fig. 2b. (**f**) RNA velocity analysis. Shown are velocity vectors projected onto the KnetL and UMAP plots from Fig. 2b, with the three Ct clusters indicated. Source data

To confirm the observed transcriptional heterogeneity of peripheral pDCs, we sorted splenic pDCs from naive B6 WT mice and analyzed them by cellular indexing of transcriptomes and epitopes by sequencing (CITE-seq). Cells from three mice were individually labeled (‘hashtagged’), stained with barcoded antibodies and subjected to single-cell RNA sequencing (scRNA-seq). After the initial clustering and the removal of a single minor tDC cluster (Extended Data Fig. 3a–d), the resulting 11,805 cells comprised seven clusters that were closely associated on a UMAP plot (Fig. 2a). To improve cluster separation, we applied K-nearest neighbor-based Network graph drawing Layout (KNetL), producing three distinct clusters (Fig. 2b) with comparably high expression of key pDC markers (Fig. 2c). Cluster 3Ct, comprising ~7% of cells in each replicate sample (Fig. 2d), was distinguished by a prominent ISG signature (Fig. 2e) that included *Mx1*, *Cd69* and *Ly6a* (encoding the surface marker Sca1) (Fig. 2f and Supplementary Table 3). Cluster 1Ct, comprising ~20% of cells in each sample, showed lower expression of *Gpm6b*, MHC class II transcripts (*H2-Ab1*, *H2-Eb1*, *H2-Dmb2*) and their master regulator *Ciita*, as well as of the antibody-derived tag (ADT) for surface MHC class II (Fig. 2e). It also showed lower ADT staining for Sca1 (Fig. 2e) along with lower expression of its transcript *Ly6a* (Fig. 2f). Apart from MHC class II and Sca1, no other ADT showed major differences between clusters (Extended Data Fig. 3e). Thus, CITE-seq confirmed the three pDC subpopulations identified by multiome snRNA-seq: a subset (~20%) with lower expression of MHC class II genes and Sca1; the majority (>70%) subset; and a small (5–7%) subset with the ISG signature. RNA velocity analysis suggested a developmental trajectory from 1Ct towards 2Ct and/or 3Ct (Extended Data Fig. 3f).

**Extended Data Fig. 4:**
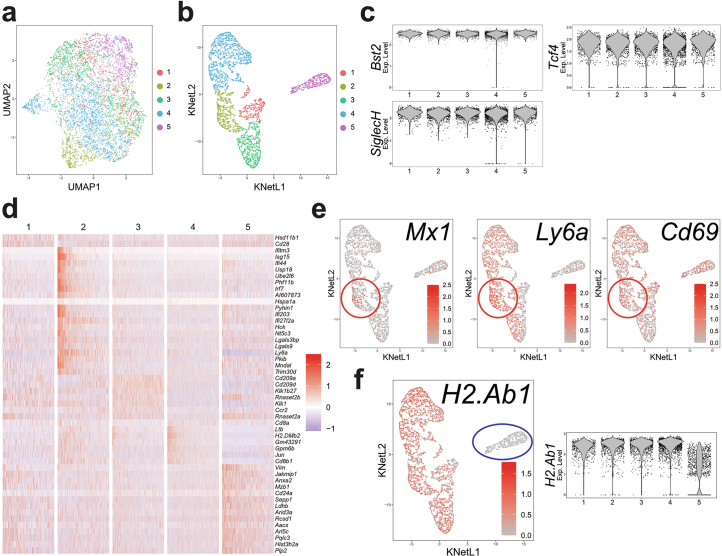
Verification of transcriptional heterogeneity in murine splenic pDCs. A previously reported scRNA-seq dataset of splenic pDCs from naive mice (GSE114313) was reanalyzed to assess steady-state heterogeneity. (**a**) UMAP plot, with clusters from the KNetL clustering in the next panel indicated by colors. (**b**) KNetL plot and clustering. (**c**) Violin plots showing transcript levels of key pDC identity genes by KNetL clusters. (**d**) Heatmap of marker gene expression for KNetL clusters. (**e**) Feature plots of key marker gene transcripts on the KNetL plot from panel B; cluster with prominent ISG expression (cluster 2) is highlighted. (**f**) The expression of MHC cl II transcript shown on the feature plot (with the MHC cl. II-low cluster 5 highlighted) and violin plot. Source data

**Extended Data Fig. 5:**
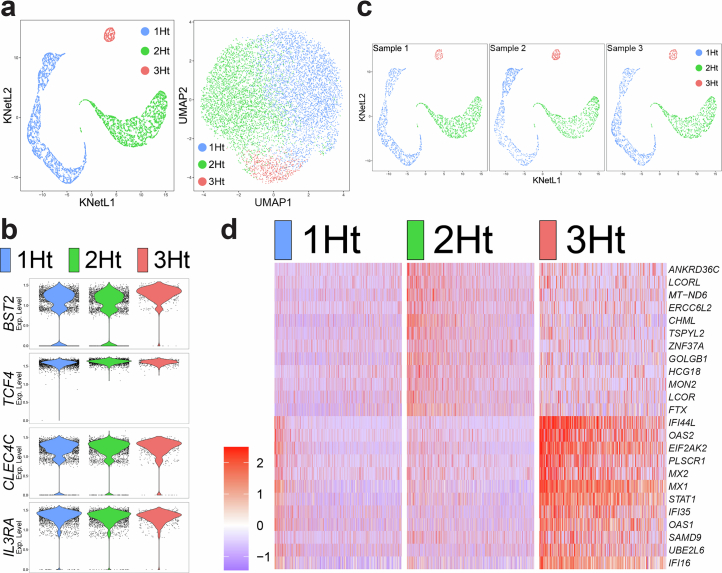
The analysis of transcriptional heterogeneity in human pDCs. A previously reported scRNA-seq dataset of pDCs from peripheral blood of three healthy human donors (GSE189120) was reanalyzed to assess steady-state heterogeneity. (**a**) KNetL and UMAP plots, with KNetL-based human transcriptome (Ht) clusters indicated. (**b**) Violin plots showing transcript levels of key pDC identity genes by KNetL clusters. (**c**) KNetL plots of Ht clusters, split up by individual donor. (**d**) Heatmap of marker gene expression for KNetL clusters. Source data

KNetL analysis of scRNA-seq of splenic pDCs from naive mice^30^ (Extended Data Fig. 4a–c) identified one cluster with an ISG signature (cluster 2, Extended Data Fig. 4d,e) and another cluster with reduced expression of *H2-Dmb2*, *Gpm6b* and *Ly6a* (cluster 5, Extended Data Fig. 4d,f), thus resembling 3Ct and 1Ct in our CITE-seq, respectively. Furthermore, we reanalyzed an scRNA-seq dataset of human pDCs from the blood of healthy donors^26^, using KNetL to resolve it into three human transcriptome clusters (Extended Data Fig. 5a,b) that were concordant between three individual donors (Extended Data Fig. 5c). Importantly, one of the clusters manifested strong IFN-I signature including *MX1* and other ISGs (Extended Data Fig. 5d), suggesting an ISG-driven heterogeneity akin to that in mouse pDCs.

We then used multiplexed immunofluorescence staining to analyze pDCs in peripheral lymph nodes from human organ donors (*n* = 5). We first identified pDCs using a combined staining for the key TFs TCF4 and IRF8 (Fig. 2g). Small fractions of these TCF4^+^IRF8^+^ pDCs showed no or high levels of MX1 protein, whereas the majority (~60%) expressed intermediate levels (Fig. 2g,h). This is consistent with the variable expression of *MX1* transcript within pDCs from blood (Fig. 2g) and suggests that human pDCs in lymphoid organs are heterogeneous with respect to the canonical ISG *MX1*. Collectively, our results suggest that mature peripheral pDCs in both mice and humans show transcriptional heterogeneity primarily driven by the ISGs.

### Prospective isolation and tracing of mouse pDC subsets

**Fig. 3:**
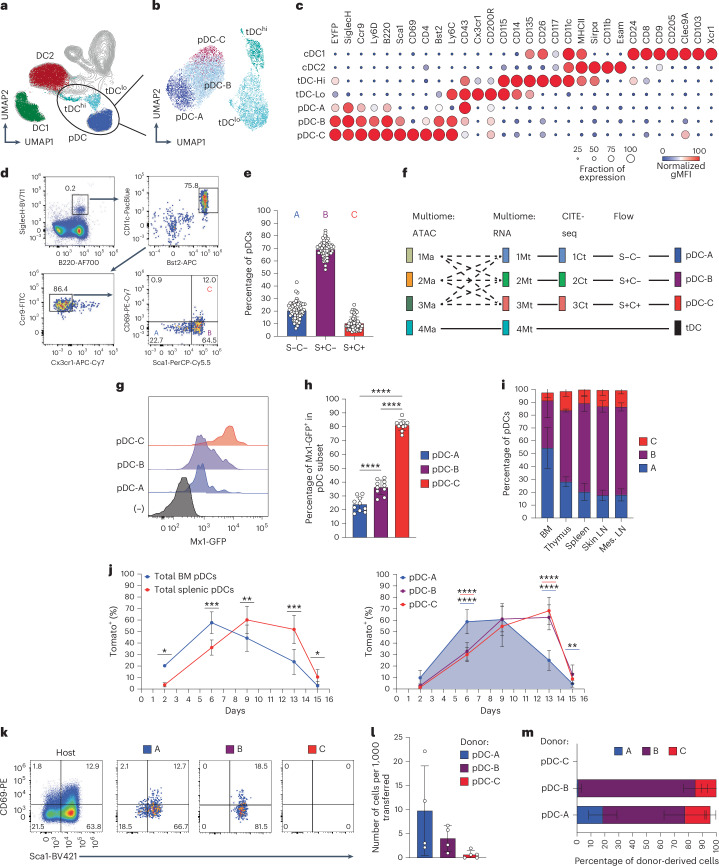
Prospective identification of pDC subsets and their differentiation trajectory. **a**–**c**, The identification of pDC subsets in the spleens of WT mice by Infinity Flow cytometry. Splenocytes from hCD2-EYFP mice were stained with 13 backbone markers followed by 1 of 39 PE-conjugated antibodies, and nonlinear dimensionality reduction was performed on Live⁺Lin^−^CD11c^+^ cells. Data shown are from one experiment. **a**, The UMAP plot of the Lin^−^CD11c^+^ fraction, highlighting the manually gated DC subsets. **b**, The UMAP plot of the extracted pDC and tDC fractions, highlighting the three subsets of pDCs (A, B and C) and the two subsets of tDCs (tDC^hi^ and tDC^lo^). **c**, The dot plot of surface marker expression in the DC subsets from **a** and **b**. **d**,**e**, The identification of pDC subsets by conventional flow cytometry. Shown is the gating scheme for splenic pDCs and a representative staining for Sca1 and CD69 in the resulting gated pDCs (**d**) and the frequency of the three phenotypic subsets within splenic pDCs from naive mice (**e**): pDC-A (Sca1^−^CD69^−^), pDC-B (Sca1^+^CD69^−^) and pDC-C (Sca1^+^CD69^+^). Bars represent means ± s.d.; symbols represent biological replicates (*n* = 60 mice) from 22 experiments. **f**, The correspondence of Ma, Mt and Ct clusters (Figs. 1 and 2) to the phenotypically defined pDC subsets. **g**,**h**, The expression of *Mx1* in pDC subsets in the *Mx1*^GFP^ reporter mouse strain. Shown are representative histograms of GFP expression in gated subsets of splenic pDCs (**g**) and the percentage of GFP^+^ cells within each subset (**h**). Bars represent means ± s.d.; symbols represent biological replicates (mice) from one experiment, representative of two experiments. **i**, Percentages of subsets within pDCs from the indicated lymphoid organs of naive WT mice. Bars represent means of biological replicates ± s.d. (BM, *n* = 45 mice; thymus, *n* = 8 mice; spleen, *n* = 68 mice; skin lymph node (LN), *n* = 8 mice; mesenteric (mes.) LN, *n* = 7 mice) from 12 experiments. **j**, The turnover of pDC subsets as determined by lineage tracing of Cx3cr1^+^ progenitors. *Cx3cr1*^CreER^ *Rosa26*^LSL−Tom^ mice were administered a single dose of tamoxifen, and tdTomato (Tom) expression within pDCs was analyzed by flow cytometry 2–15 d later. Shown is the fraction of Tom^+^ cells in total pDCs in the BM and spleen (left) or in the splenic pDC subsets (right). Symbols represent means of biological replicates ± s.d. (day 2, *n* = 2 mice; day 6, *n* = 10 mice; day 9, *n* = 9 mice; day 13, *n* = 8 mice; day 15, *n* = 5 mice) from 4 experiments. Significant differences between pDC-A and pDC-B (blue bars) or pDC-A and pDC-C (red bars) are indicated. **k**–**m**, In vivo differentiation potential of pDC subsets. pDC subsets were sorted from the spleens of *Cx3cr1*^GFP^ CD45.1 mice to exclude GFP^+^ tDCs and adoptively transferred into naive congenic WT CD45.2 mice, and splenic pDCs were analyzed 2 d later. Shown are the phenotypes (**k**), numbers (**l**) and subset distribution (**m**) of CD45.2^+^ donor-derived pDCs. Bars represent means ± s.d.; symbols represent biological replicates (host and donor mouse pairs) from four experiments. Statistical significance was analyzed using paired one-way ANOVA followed by Tukey’s test (**h** and **j**, right), or paired two-sided Student’s *t*-test (**j**, left); *P < 0.05; **P < 0.005; ***P < 0.0005; ****P < 0.00005. Source data

**Extended Data Fig. 6:**
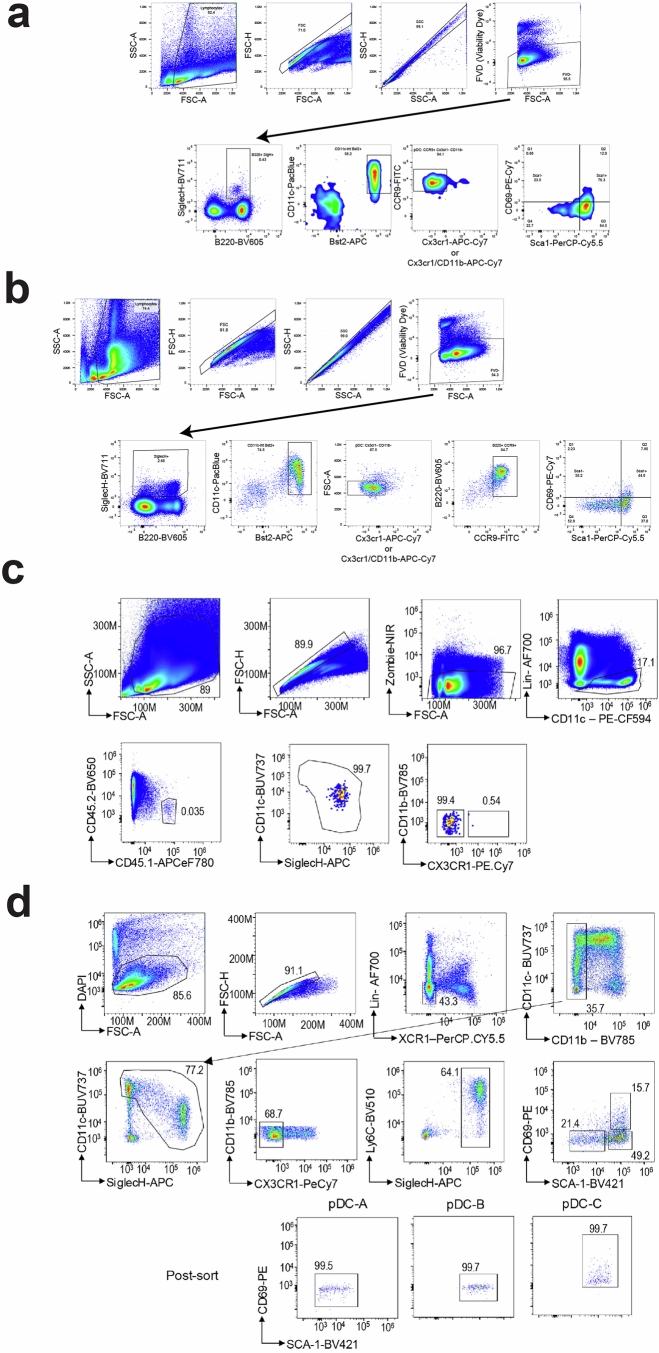
Prospective identification and isolation of murine pDCs. (**a**-**b**) Gating strategy for the analysis of pDC subsets in the spleen (panel **a**) and bone marrow (panel **b**). (**c**) Gating strategy for the recovery of pDC subsets in the hosts of adoptive transfer experiments in Fig. 3k-m. (**d**) Gating strategy for the sorting (top panel) and post-sort purity (bottom panel) of pDC subsets isolated for functional experiments in Figs. 7–8. Source data

To prospectively isolate the newly identified pDC subsets, we first used Infinity Flow to assay the expression of 39 different markers across mouse splenic dendritic cell (DC) populations. We applied it to *hCD2*-Cre mice harboring a Cre-inducible enhanced yellow fluorescent protein (EYFP) (*Rosa26*^LoxStopLox-EYFP^), in which EYFP is expressed in lymphocytes, pDCs and tDCs, but not cDCs^31,32^. Within the splenic CD11c^+^ population, major DC subsets including pDCs, cDCs and tDCs, including the pDC-like CD11c^lo^ (tDC^lo^) and the cDC-like CD11c^hi^ (tDC^hi^) tDC subsets^10^, could be identified (Fig. 3a). pDCs and tDCs were clearly separable by multiple markers, in particular by SiglecH on pDCs versus Cx3cr1 on tDCs (Fig. 3a–c). Within pDCs, three subsets (tentatively designated A, B or C) could be distinguished based on a combination of Sca1 and CD69 (Fig. 3c). The pDC-A subset manifested relatively lower levels of pDC-specific markers such as Ccr9, Ly-6D and Bst2; however, they were still >50% positive compared with the virtually negative tDCs (Fig. 3c). Accordingly, conventional flow cytometry of gated splenic pDCs (Extended Data Fig. 6a,b) revealed three subsets: Sca1^−^CD69^−^, Sca1^+^CD69^−^ and Sca1^+^CD69^+^ (Fig. 3d), comprising ~20%, 70% and 10%, respectively (Fig. 3e). By both frequency and phenotype, the three subsets corresponded to clusters 1, 2 and 3 of the Mt (Fig. 1) and Ct (Fig. 2) datasets, suggesting a common nomenclature of steady-state pDCs: the ‘naive’ subset, pDC-A (Sca1^−^CD69^−^); the majority subset expressing Sca1, pDC-B (Sca1^+^CD69^−^); and the minor subset expressing the full ISG signature, pDC-C (Sca1^+^CD69^+^) (Fig. 3f). As further validation, we used the fluorescent reporter strain (*Mx1*^GFP^) for the canonical ISG *Mx1*. Indeed, pDC-A was largely GFP^−^; pDC-B showed a minor but significant upregulation of GFP; and pDC-C was >80% GFP^+^ (Fig. 3g,h). We conclude that our staining scheme captures ISG-driven transcriptional heterogeneity of pDCs.

BM harbored the highest proportion of pDC-A and the lowest fraction of pDC-B and pDC-C among lymphoid tissues (Fig. 3i), suggesting that these subsets represent a developmental progression. We tested this using lineage tracing in *Cx3cr1*^CreER^ *Rosa26*^LoxStopLox-Tom^ mice, in which tamoxifen-induced Cre recombination labels *Cx3cr1-*expressing DC progenitors with the Cre-induced fluorescent protein tdTomato (Tom)^32^. The labeling of total pDCs in the BM and spleen peaked at ~60% of Tom^+^ cells on days 6 and 9, respectively, consistent with the migration of mature pDCs from BM into the periphery (Fig. 3j). Within splenic pDCs, the labeling of pDC-A reached a plateau at ~60% on days 6–9 and subsequently declined by day 15. The labeling of pDC-B and pDC-C reached the same levels on days 9–13 followed by a sharp decline (Fig. 3j), suggesting that the pDC-A subset arises earlier than pDC-B and pDC-C. To directly test the relationships between pDC subsets, we sorted them from the spleens of *Cx3cr1*^GFP^ CD45.1 mice (thus avoiding any contamination with GFP^hi^ tDCs), adoptively transferred them into unmanipulated B6 CD45.2 mice and recovered them from recipient spleens 2 d later (Extended Data Fig. 6c). pDC-A gave rise to all pDC subsets at the usual ratios; pDC-B produced only pDC-B and pDC-C; and pDC-C could not be recovered (Fig. 3k–m). Thus, the three subsets represent a progression from the least mature (pDC-A) to terminally differentiated (pDC-C).

### Tonic IFN-I signaling drives steady-state pDC heterogeneity

**Fig. 4:**
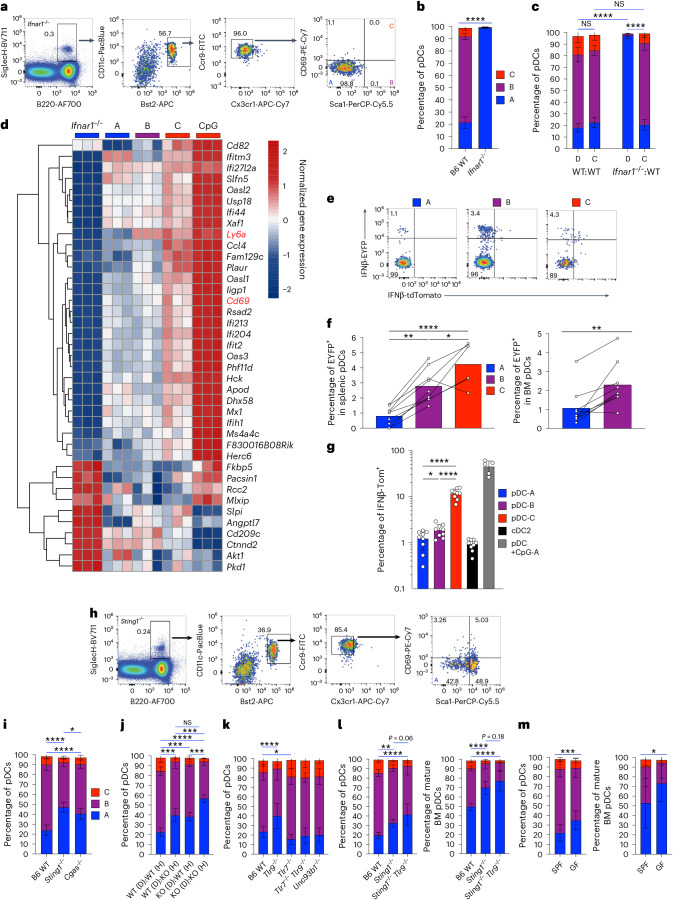
pDC heterogeneity is generated by tonic IFN-I signaling that partially depends on cGAS/STING and TLR9 signaling. **a**,**b**, pDC heterogeneity in IFNAR-deficient mice. Shown are the gating scheme and a representative staining for Sca1 and CD69 in splenic pDCs from a naive *Ifnar1*^−/−^ mouse (**a**) and the fractions of pDC subsets in B6 WT versus *Ifnar1*^−/−^ mice (**b**). Bars represent means of biological replicates ± s.d. (B6 WT, *n* = 6 mice; *Ifnar1*^−/−^, *n* = 5 mice) from 2 experiments. Significance of differences between pDC-A subsets is indicated. **c**, Cell-intrinsic role of IFNAR signaling in pDC heterogeneity. BM from WT or *Ifnar1*^−/−^ mice (CD45.2^+^) was mixed 1:1 with the BM from WT competitor (CD45.1^+^) mice and transferred into irradiated CD45.1^+^ recipients. Shown are the fractions of pDC subsets within donor (D)- or competitor (C)-derived splenic pDCs from recipients of WT:WT or *Ifnar1*^−/−^:WT BM. Bars represent means of biological replicates ± s.d. (WT:WT, *n* = 7 mice; *Ifnar1*^−/−^:WT, *n* = 4 mice) from 2 experiments. Significance of differences between pDC-A subsets is indicated. **d**, Transcriptome analysis of prospectively isolated pDC subsets. pDC-A/B/C subsets defined by Sca1 and CD69 expression were sorted from splenocytes of three individual naive WT mice and analyzed by bulk RNA-seq. Total pDCs from *Ifnar1*^−/−^ mice (phenotypic pDC-A) and from WT mice 12 h after CpG-A administration (phenotypic pDC-C) were sorted and analyzed in parallel (three separate mice per condition). Shown is the heatmap of top differentially expressed transcripts across individual samples. **e**–**g**, The history of *Ifnb1* gene expression in pDC subsets as determined by lineage tracing in *Ifnb1*^tdTom-iCre^ *Rosa26*^LoxStopLox-EYFP^ reporter mice. **e**, Representative plots showing expression of EYFP and Tom in gated subsets of splenic pDCs from naive reporter mice. **f**, Percentage of EYFP^+^ cells within pDC subsets in the spleen (left) and BM (right). Bars represent means, symbols represent biological replicates (mice) from two experiments and lines connect paired samples from the same mouse. **g**, Percentage of Tom^+^ cells within pDC subsets in the spleen with groups ‘cDC2’ and ‘pDC+CpG-A’ shown as controls. Bars represent means ± s.d.; symbols represent biological replicates (mice) from one experiment, representative of three experiments. **h**, Representative gating scheme and staining for Sca1 and CD69 in splenic pDCs from a naive S*ting1*^−/−^ mouse. **i**, The fractions of pDC subsets in B6 WT, *Sting1*^−/−^ or *Cgas*^−/−^ mice. Bars represent means of biological replicates ± s.d. (B6 WT, *n* = 11 mice; *Sting1*^−/−^, *n* = 9 mice; *Cgas*^−/−^, *n* = 8 mice) from 4 experiments. Significance of differences between pDC-A subsets is indicated. **j**, Total BM from WT or S*ting1*^−/−^ knockout (KO) donor mice was transferred into irradiated WT or S*ting1*^−/−^ host mice. Shown is the fraction of pDC subsets within splenic pDCs in the resulting chimeric mice with the indicated donor (D) and host (H) genotypes. Bars represent means of biological replicates ± s.d. (WT (D):WT (H), *n* = 7 mice; WT (D):KO (H), *n* = 5 mice; all else, *n* = 4 mice) from 1 experiment. Significance of differences between pDC-A subsets is indicated. **k**, Fractions of pDC subsets within total splenic pDCs in mice with null alleles of the indicated TLR pathway genes. Bars represent means of biological replicates ± s.d. (B6 WT, *n* = 20 mice; *Tlr9*^−/−^, *n* = 13 mice; *Tlr7*^−/−^, *n* = 19 mice; *Tlr7*^−/−^*Tlr9*^−/−^, *n* = 14 mice; *Unc93b1*^−/−^, *n* = 11 mice) from 12 experiments. Significance of differences between pDC-A subsets is indicated. **l**, Fractions of pDC subsets within total splenic (left) or BM (right) pDCs in mice deficient for *Sting1* alone or with *Tlr9*. Bars represent means of biological replicates ± s.d. (*Sting1*^−/−^*Tlr9*^−/−^, *n* = 6 mice; all else, *n* = 8 mice) from 2 experiments. Significance of differences between pDC-A subsets is indicated. **m**, Fractions of pDC subsets within total splenic (left) or BM (right) pDCs in SPF or GF B6 WT mice. Bars represent means of biological replicates ± s.d. (SPF spleen, *n* = 15 mice; GF spleen, *n* = 23 mice; SPF BM, *n* = 11 mice; GF BM, *n* = 20 mice) from 6 experiments. Significance of differences between pDC-A subsets is indicated. Statistical significance was analyzed using unpaired two-sided Student’s *t*-test (**b**,**m**), paired two-sided Student’s *t*-test (**f** (right)), one-way ANOVA followed by Tukey’s test (**c**,**f** (left),**i**–**l**) or paired one-way ANOVA followed by Tukey’s test (**g**); *P < 0.05; **P < 0.005; ***P < 0.0005; ****P < 0.00005. Comparisons with control omitted from statistical analysis: gray bar (**g**). NS, not significant. Source data

Considering the ISG signature found in steady-state pDCs, we interrogated the role of IFN-I signaling in pDC heterogeneity. Splenic pDCs from mice deficient in IFNAR (*Ifnar1*^−/−^) manifested a complete loss of Sca1 and CD69 and thus phenotypically corresponded to pDC-A (Fig. 4a,b). In mixed chimeras generated from naive WT or *Ifnar1*^−/−^ mice, *Ifnar1*^−/−^ splenic pDCs comprised exclusively pDC-A while pDCs from competitor WT BM retained heterogeneity (Fig. 4c), demonstrating a cell-intrinsic role of IFN-I signaling. To further explore the ISG signature within pDC subsets, we performed bulk RNA sequencing (RNA-seq) on sorted splenic pDCs from individual naive mice (*n* = 3) that were WT (pDC-A/B/C) or *Ifnar1*^−/−^ (total pDCs comprising phenotypic pDC-A). We also isolated total pDCs from WT mice challenged in vivo with CpG, in which all pDCs upregulated Sca1 and CD69 and phenotypically resembled pDC-C. As expected, *Cd69* was detected in pDC-C samples and *Ly6a* was high in both pDC-B and pDC-C, providing the best discrimination between these subsets and pDC-A (Fig. 4d and Supplementary Table 4). The ISG signature was the strongest in pDC-C from CpG-challenged mice but was still apparent in pDC-C from naive WT mice, whereas it was low in pDC-A/B subsets and absent in *Ifnar1*^−/−^ pDCs (Fig. 4d). Of note, the transcript for the key IFN-I inducing TF *Irf7* was reduced in *Ifnar1*^−/−^ pDCs but expressed at high levels in both pDC-A and pDC-B; as an ISG, it was further elevated in pDC-C (Supplementary Table 4) as also observed in single-cell profiling (Fig. 1d). Thus, the transcriptional and phenotypic pDC heterogeneity in the steady state is generated by IFN-I signaling.

To test whether some of the relevant IFN-I might be produced by pDCs themselves, we crossed mice in which tdTomato and Cre recombinase are expressed from the *Ifnb1* locus (*Ifnb1*^tdTom-iCre^)^33^ to *Rosa26*^LoxStopLox-EYFP^ mice. In the resulting reporter mice, the expression levels of Tom and EYFP reflect the current and previous expression of *Ifnb1*, respectively. Splenic pDC-A contained <1% EYFP^+^ cells, and this fraction significantly rose to ~3% in pDC-B and to ~4% in pDC-C (Fig. 4e,f). A similarly significant increase in EYFP expression between pDC-A and pDC-B was also observed in the BM (Fig. 4f). Moreover, Tom was expressed at background levels (~1%) in pDC-A, increased to ~2% in pDC-B and reached ~10% in pDC-C (Fig. 4g), suggesting an ongoing expression of *Ifnb1* in the latter.

We explored the innate sensing pathways that drive tonic IFN-I production and pDC heterogeneity. The frequency of pDC-A was significantly increased in mice deficient for STING or cGAS, suggesting a role of the cGAS/STING-induced IFN-I production (Fig. 4h,i). The minor (if significant) difference between the two models may reflect the role of STING as an integrator of multiple signaling pathways. In reciprocal BM transfers between naive WT and *Sting1*^−/−^ mice, pDCs derived from *Sting1*^−/−^ donors and *Sting1*^−/−^ hosts showed a >2-fold increase in pDC-A (Fig. 4j). Chimeras lacking STING signaling in either host or donor cells showed intermediate pDC-A frequencies (Fig. 4j), suggesting that cGAS/STING signaling in both hematopoietic and nonhematopoietic cells contributes to IFN-I-driven pDC diversification. Next, we tested the role of endosomal TLRs that drive IFN-I production by pDCs and other immune cell types. Whereas *Tlr9*^−/−^ mice exhibited elevated splenic pDC-A frequency, *Tlr7*^−/−^ mice showed a significant contraction of pDC-A and expansion of pDC-C (Fig. 4k). The deletion of both receptors in *Tlr7*^−/−^*Tlr9*^−/−^ mice, or their functional impairment in mice deficient for their common trafficking chaperone *Unc93b1*, phenocopied *Tlr7*^−/−^ mice (Fig. 4k). Thus, TLR9 contributes to IFN-I-driven phenotypic diversification into pDC-B/C, whereas TLR7 diminishes it and is genetically dominant over TLR9, reflecting a well-established antagonism between these receptors^34^. *Sting1*^−/−^*Tlr9*^−/−^ mice did not show a significant increase in pDC-A over *Sting1*^−/−^ mice (Fig. 4l), suggesting that the two pathways act in parallel, but not synergistically, to drive pDC heterogeneity.

Because commensal microbiota was shown to affect pDC homeostasis^24^ and induce tonic IFN-I signaling in pDCs^35^ and other cells^36,37^, we tested its role using germ-free (GF) mice. Compared with conventional specific pathogen-free (SPF) mice, GF mice exhibited a significant expansion of pDC-A in both spleen and BM (Fig. 4m). Thus, pDC heterogeneity is generated in part by the commensal microbiota, which activate the cGAS/STING and/or TLR9 pathways to elicit tonic IFN-I signaling.

### pDC heterogeneity correlates with cytokine-producing capacity

**Fig. 5:**
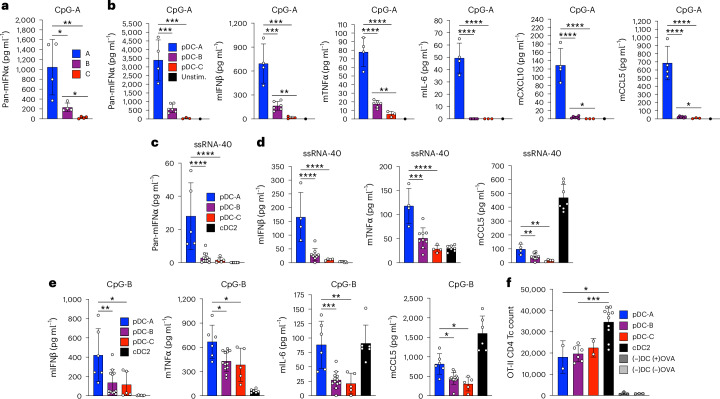
pDC heterogeneity correlates with distinct cytokine-producing capacity. **a**–**e**, Phenotypic pDC subsets were sorted from the splenocytes pooled from six naive WT mice and cultured (2 × 10^4^ per well) in the presence of TLR agonists, and concentrations of indicated proteins in the supernatants were measured 12–18 h later. For each multiplex panel, all analytes were measured at the same time point. In all panels, bars represent means ± s.d., and symbols represent values from independently sorted biological replicates (single cultures or averages of technical replicates). Note that the rare pDC-A and pDC-C populations were assayed in fewer experiments than for pDC-B. **a**, Cells were stimulated with CpG-A and IFNα was measured by ELISA. **b**, Cells were stimulated with CpG-A and indicated analytes were measured by a multiplex bead array. **c**, Cells were stimulated with ssRNA40 and IFNα was measured by ELISA. **d**, Cells were stimulated with ssRNA40 and indicated analytes were measured by a multiplex bead array. **e**, Cells were stimulated with CpG-B and indicated analytes were measured by a multiplex bead array. **f**, T cell priming by pDC subsets. pDC subsets or CD11c^hi^CD11b^+^ cDC2 were sorted from pooled spleens of naive B6 WT mice, plated at 3 × 10^4^ per well and co-cultured with 3 × 10^4^ magnetically enriched T cells from OT-II mice in the presence of OVA_323–339_ peptide. The numbers of OVA-specific (TCR-Vβ-5.1/2^+^) CD4^+^ T cells in each well were determined by flow cytometry 64 h later. Control wells were seeded with T cells without DCs with or without OVA. Bars represent means ± s.d.; symbols represent cultures of separately sorted cells from one experiment, representative of three experiments. Statistical significance was analyzed using one-way ANOVA followed by Tukey’s test; *P < 0.05; **P < 0.005; ***P < 0.0005; ****P < 0.00005. Statistics for comparisons with controls not shown: black bars (**b**–**e**), gray bars (**f**). Comparisons with control omitted from statistical analysis: black bar (**b**). m, mouse; Tc, T cell; Unstim., unstimulated. Source data

To explore potential functional differences between the identified pDC subsets, we sorted them from spleens of naive WT mice and measured stimulation-induced cytokine secretion in vitro. Upon TLR9 stimulation with CpG-A, pDC-A secreted the highest amount of IFNα, IFNβ, inflammatory mediators (TNF and IL-6) and chemokines (CCL5 and CXCL10) (Fig. 5a,b). pDC-B produced lower but detectable amounts of IFN-I and other mediators, whereas pDC-C produced little to none. Similar results were obtained with the TLR7 agonist ssRNA40 (Fig. 5c,d). Control cDCs failed to produce IFN-I, although they produced detectable TNF and high levels of CCL5 (Fig. 5c,d). Upon TLR9 stimulation with CpG-B, which elicits NF-κB-dependent pro-inflammatory signaling, pDC-A still produced more IFNβ, TNF, IL-6 and CCL5, although the difference was smaller than for CpG-A (Fig. 5e). In contrast, all pDC subsets showed comparable ability to present MHC class II-restricted peptide to CD4^+^ T cells (Fig. 5f). Thus, pDC subsets manifest differential cytokine-producing capacities that inversely correlate with their IFN-I ‘experience’.

### Signals that drive pDC heterogeneity modulate pDC function

**Fig. 6:**
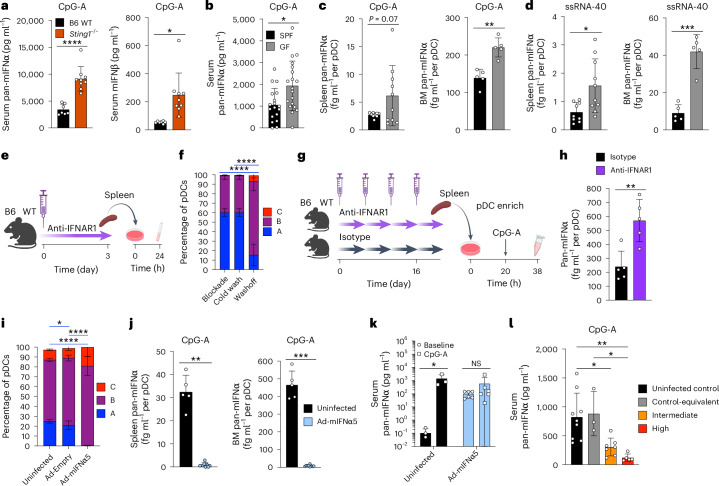
pDC heterogeneity and function are dynamically modulated by IFN-I signaling. **a**, In vivo pDC function in STING-deficient mice. Naive B6 WT (*n* = 7) or *Sting1*^−/−^ (*n* = 9) mice were injected with CpG-A, and serum IFNα and IFNβ were measured by ELISA 6 h later. Bars represent means ± s.d.; symbols represent biological replicates (mice) from one experiment, representative of three experiments. **b**–**d**, pDC function in SPF versus GF mice. **b**, pDC-mediated response in vivo. WT SPF (*n* = 18) or GF (*n* = 19) mice were injected with CpG-A, and serum IFNα was measured by ELISA 6 h later. Bars represent means ± s.d.; symbols represent biological replicates (mice) from three experiments. **c**,**d**, pDC-mediated response in vitro. DC-enriched fraction of splenocytes or total BM cells from SPF or GF mice were stimulated with CpG-A (**c**) or ssRNA40 (**d**), and IFNα was measured in the supernatant by ELISA 18 h later. The fraction of pDCs in each sample was measured in parallel by flow cytometry and used to calculate IFNα amount per seeded pDC. Bars represent means ± s.d.; symbols represent biological replicates (mice) from two experiments (spleen, total *n* = 9 per condition) or one experiment (BM, *n* = 5 per condition). **e**–**h**, The effect of transient IFN-I blockade on pDC heterogeneity and function. **e**,**f**, B6 WT mice were injected with anti-IFNAR1 mAb, and splenic pDCs were analyzed by flow cytometry ex vivo, after wash with cold media, or after culture of splenocytes for 24 h (washoff). **e** shows the experimental schematic. **f** shows the fractions of subsets within pDCs. Bars represent means ± s.d. (*n* = 5 mice) from 1 experiment, representative of 2 experiments. Significance of differences between pDC-A subsets is indicated. **g**,**h**, WT mice were injected with anti-IFNAR1 mAb over 20 d, and splenic pDCs were enriched by magnetic sorting, cultured for 20 h and stimulated with CpG-A for 18 h. **g** shows the experimental schematic. **h** shows IFNα concentrations in the supernatants per pDC as described in **c** and **d**. Bars represent means ± s.d.; symbols represent biological replicates (mice) from one experiment. **i**–**l**, The effect of constitutive IFN-I signaling on pDC heterogeneity and function. B6 WT mice were left uninfected or administered Ad-mIFNα5; an empty vector (Ad-Empty) was used as a control. **i**, Fractions of subsets within splenic pDCs 6 weeks after Ad-mIFNα5 administration. Bars represent means of biological replicates ± s.d. (Ad-mIFNα5, *n* = 14 mice; all else, *n* = 5 mice) from 3 experiments. Significance of differences between pDC-A subsets is indicated. **j**, IFN-I production in vitro. DC-enriched fraction of splenocytes or total BM cells from mice 6 weeks post administration were stimulated with CpG-A, and IFNα was measured in the supernatant by ELISA 18 h later. Data represent IFNα per seeded pDC as above. Bars represent means ± s.d.; symbols represent biological replicates (uninfected, *n* = 5 mice; Ad-mIFNα5, *n* = 9 mice) from 2 experiments. **k**, IFN-I production in vivo. Mice 6 weeks post administration were injected with CpG-A, and serum IFNα was measured by ELISA before (baseline) or 6 h after CpG-A injection. Bars represent means ± s.d.; symbols represent biological replicates (mice) from one experiment. **l**, IFN-I production in vivo in mice with different levels of IFN-I signaling. Mice 18 weeks post administration were analyzed for the expression of Sca1 on peripheral blood lymphocytes, and grouped according to the expression levels equivalent to controls, intermediate or high (corresponding to absent, low and high IFN-I, respectively). After 3 d, mice were injected with CpG-A, and serum IFNα was measured by ELISA 6 h later. Bars represent means ± s.d.; symbols represent biological replicates (mice) from one experiment. Statistical significance was analyzed using one-way ANOVA followed by Tukey’s test (**f**,**i**,**l**), ratio paired two-sided Student’s *t*-test (**k**) or unpaired two-sided Student’s *t*-test (other panels). *P < 0.05; **P < 0.005; ***P < 0.0005; ****P < 0.00005. Illustrations in **e** and **g** created using BioRender.com. Source data

We tested whether the in vivo modulation of pDC subset composition would affect their function. The administration of CpG-A, which elicits IFN-I production exclusively from pDCs^7,38^, resulted in higher IFN-I concentrations in the sera of *Sting1*^−/−^ mice (Fig. 6a). Similarly, GF mice showed higher serum IFNα than SPF mice after CpG-A challenge (Fig. 6b). Upon TLR9 stimulation in vitro, BM pDCs from GF mice produced significantly more IFNα, while splenic pDCs showed a similar (albeit not significant) trend (Fig. 6c). Likewise, splenic and BM pDCs from GF mice produced significantly more IFNα upon TLR7 stimulation (Fig. 6d). These data on pDC-driven responses contrast with lower IFN-I production by GF mice after poly-I:C challenge^39^ or during alphavirus infection^40^, suggesting that the effect of commensal microbes on IFN responses is context-specific. Overall, blocking some of the stimuli (microbiota) or sensors (STING) of tonic IFN-I production expanded the pDC-A subset and enhanced pDC-dependent responses.

**Extended Data Fig. 7:**
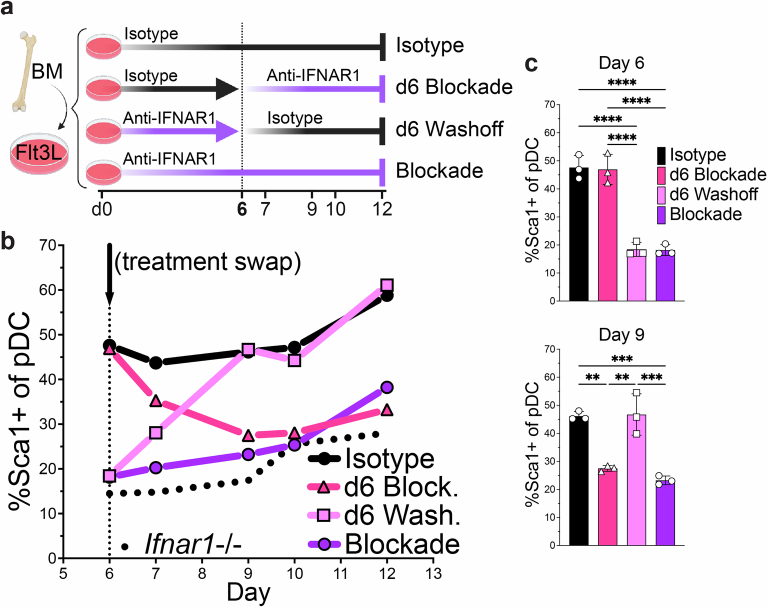
Modulation of pDC heterogeneity in bone marrow-derived pDCs by IFN-I blockade. (**a**) Total BM cells were harvested from naïve wild-type mice and cultured in the presence of Flt3L for 12 days to induce DC differentiation. Anti-IFNAR1 mAb was added throughout the culture (Blockade), during the first 6 days (D6 washoff) or during the last 6 days (D6 blockade). (**b**) The dynamics of Sca1 expression on pDCs between days 6 and 12 of culture, as determined by flow cytometry. Cultures from *Ifnar1*^−/−^ mice were used as controls (dashed line). Data represent averages of biological replicates (cultures from individual mice) from one experiment. (**c**) The fraction of Sca1^+^ pDCs on days 6 and 9 of culture. Bars represent means ± SD, symbols represent biological replicates (cultures from individual mice) from one experiment. Statistical significance was analyzed using one-way ANOVA followed by Tukey’s test. * p < 0.05; ** p < 0.005; *** p < 0.0005; **** p < 0.00005. Illustration in **a** created using BioRender.com. Source data

To further test the role of tonic IFN-I signaling, we transiently blocked it using anti-IFNAR1 monoclonal antibody (mAb). The addition of anti-IFNAR1 mAb during the entire 12 d abolished Sca1 expression (Extended Data Fig. 7b). The addition of the mAb for the last 6 d similarly inhibited Sca1 expression, whereas the removal of the mAb after 6 d restored Sca1 expression to normal levels (Extended Data Fig. 7b,c). Thus, some IFN-I signaling that drives pDC heterogeneity is occurring during pDC development in vitro, and can be reversibly blocked by anti-IFNAR1 mAb.

The treatment of naive WT mice with a single dose of anti-IFNAR1 (Fig. 6e) induced the expansion of pDC-A (~60%) and loss of pDC-C in the splenic pDCs (Fig. 6f). However, the 24-h culture without anti-IFNAR1 restored the normal subset distribution, confirming that the latter is driven by an ongoing IFN-I signaling. The loss of IFN-I signaling can profoundly impair the IFN-I response of pDCs, depending on the pathophysiological context^19,38,41^. Therefore, to test the consequences of the blocked tonic IFN-I signaling, IFNAR blockade had to be reversed before activation. We treated mice with anti-IFNAR mAb over 16 d, isolated total splenocytes, cultured them for 20 h to reverse IFNAR blockade, stimulated with CpG and measured IFNα secretion (Fig. 6g). We found that pDCs from anti-IFNAR1-treated mice produced significantly more IFNα than those from isotype control-treated mice (Fig. 6h). Overall, transient blockade of IFN-I signaling enhances IFN-I production by pDCs, consistent with its role in the functional diversification of pDC subsets.

We tested the effect of enforced IFN-I signaling by using the adenoviral vector encoding mouse IFNα5 (Ad-mIFNα5), which drives systemic IFNα production by hepatocytes for 4–12 weeks followed by a gradual decline. At the peak of IFNα production (6–7 weeks after injection), mice administered Ad-mIFNα5, but not a control adenovirus, completely lacked pDC-A (Fig. 6i). Notably, the pDC-C subset was not expanded, suggesting that exogenous IFN-I may be insufficient for their generation. Furthermore, splenocytes and BM cells from Ad-mIFNα5-treated mice failed to produce IFNα when cultured with CpG-A (Fig. 6j). While control mice showed strong induction of IFNα by CpG-A challenge in vivo, Ad-mIFNα5-treated mice showed detectable baseline levels but no additional induction of IFNα (Fig. 6k). Finally, we examined Ad-mIFNα5-treated mice at 16 weeks, that is, when IFNα levels decline. Using Sca1 expression on blood leukocytes as a proxy for IFNα activity, we divided mice into those with negative, intermediate or high IFN-I activity. Upon challenge with CpG-A, control untreated mice and those with negative (control-equivalent) IFN-I activity showed high IFNα response, whereas those with intermediate and high IFN-I activity showed progressively decreasing IFNα response (Fig. 6l). Thus, exogenous IFN-I induces a shift towards pDC-B/C and profoundly impairs their IFN-I-producing capacity.

### pDC heterogeneity underlies susceptibility to virus infection

**Fig. 7:**
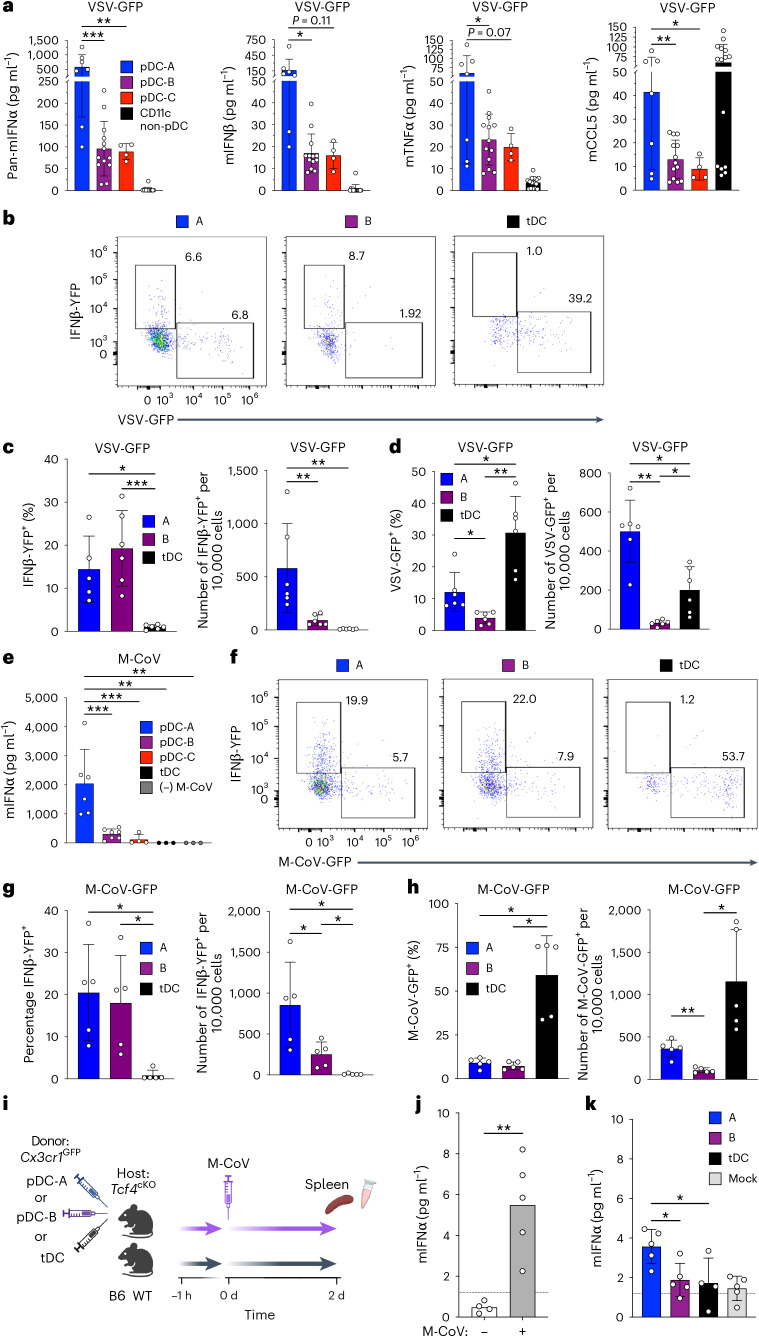
pDC heterogeneity correlates with distinct responses and susceptibility to virus infection. **a**, Cytokine response of pDC subsets to VSV infection. Splenic pDC subsets or cDC2s were sorted from B6 WT mice and cultured with VSV-GFP for 18 h, and indicated cytokines in the supernatants were measured by multiplex bead array. Bars represent means ± s.d.; symbols represent biological replicates (cultures from individual mice) from two experiments. **b**–**d**, Single-cell response of pDC subsets to VSV. Splenic pDC-A and pDC-B subsets or tDCs were sorted from *Ifnb1*^EYFP^ mice, cultured with VSV-GFP for 8 h and analyzed by flow cytometry. **b**, Representative flow cytometry plots of GFP and YFP fluorescence. **c**,**d**, The fraction and absolute numbers of *Ifnb1*-expressing EYFP^+^ cells (**c**) and of virus-infected GFP^+^ cells (**d**) in each subset. Bars represent means ± s.d.; symbols represent biological replicates (cultures from individual mice) from five experiments. **e**, Cytokine response of pDC subsets to M-CoV infection. Splenic pDC subsets or tDCs were sorted from B6 WT mice and cultured with M-CoV-GFP for 14 h, and IFNα in the supernatants was measured by ELISA. Bars represent means ± s.d.; symbols represent biological replicates (cultures from individual mice) from two experiments. **f**–**h**, Single-cell response of pDC subsets to M-CoV. Splenic pDC-A and pDC-B subsets or tDCs were sorted from *Ifnb1*^EYFP^ mice, cultured with M-CoV-GFP for 8 h and analyzed by flow cytometry. **f**, Representative flow cytometry plots of GFP and YFP fluorescence. **g**,**h**, The fraction and absolute numbers of *Ifnb1*-expressing EYFP^+^ cells (**g**) and of virus-infected GFP^+^ cells (**h**) in each subset. Bars represent means ± s.d.; symbols represent biological replicates (cultures from individual mice) from four experiments. **i**,**j**, The analysis of IFN-I production by pDC subsets in vivo. **i**, The experimental scheme. Donor *Cx3cr1*^*e*GFP^ reporter mice were used to sort splenic pDC-A or pDC-B subsets (GFP^−^) or tDCs (GFP^+^), which were transferred into pDC-deficient *Tcf4*^flox/flox^ *Cd11c*-Cre conditional knockout (Tcf4^cKO^) hosts. After 1 h, hosts were infected with M-CoV, and IFNα was measured 2 d later by ELISA of spleen tissue lysates. **j**, The detection of IFNα in the spleen lysates of control B6 WT mice that were uninfected or infected 2 d earlier with M-CoV. The limit of IFNα detection in undiluted spleen lysates is indicated by a dotted line. Bars represent means ± s.d.; symbols represent biological replicates (mice) from one experiment. **k**, IFNα concentration in the spleen lysates of M-CoV-infected Tcf4^cKO^ recipient mice that were adoptively transferred with pDC fractions or tDCs, or had mock transfer (as described in **i**). Bars represent means ± s.d.; symbols represent biological replicates (mice, each host receiving donor cells from separate host mice) from four experiments. Statistical significance was analyzed using one-way ANOVA followed by Tukey’s test. *P < 0.05; **P < 0.005; ***P < 0.0005. Illustration in **i** created using BioRender.com. Source data

To confirm the differential functionality of pDC subsets (Fig. 5) during virus-induced activation, pDC subsets from naive WT mice were cultured with live VSV encoding GFP (VSV-GFP). As with synthetic TLR ligands, pDC-A produced significantly more IFNα than other subsets (Fig. 7a); a similar trend was also observed for IFNβ, TNF and CCL5. Importantly, pDC-B and pDC-C secreted lower but detectable levels of these mediators, in contrast to cDCs which secreted no IFN-I or TNF but high CCL5.

We then tested the IFN-I response of pDCs at the single-cell level using the reporter mice expressing YFP from the *Ifnb1* locus (*Ifnb*^mob^, ref. ^17^). We used splenocytes from *Ifnb*^mob^ mice to sort pDC-A and pDC-B (the numbers of pDC-C were too low), as well as tDCs for comparison. Sorted cells were cultured with VSV-GFP, and *Ifnb1* expression and virus replication were assayed 8 h later by spectral flow cytometry for EYFP and GFP, respectively. Nearly all EYFP^+^ cells were negative for GFP (Fig. 7b), consistent with a previous report that IFN-I-producing pDCs are not infected with VSV^29^. Both pDC-A and pDC-B harbored a comparable proportion (15–18%) of EYFP^+^ cells; however, pDC-A yielded a significantly higher absolute number of these cells (Fig. 7c), mirroring their higher net production of IFN-I by enzyme-linked immunosorbent assay (ELISA) (Fig. 7a). Notably, pDC-A also harbored significantly more VSV-infected GFP^+^ cells, both as a fraction and as absolute numbers (Fig. 7d). In contrast, tDCs harbored almost no EYFP^+^ cells and the highest fraction of GFP^+^ cells (Fig. 7c,d), confirming the inability of tDCs to secrete IFN-I (ref. ^10^) and revealing their heightened susceptibility to infection.

We extended this analysis to M-CoV, a coronavirus that activates pDCs to produce IFN-I in vivo^5,6,10,42^. Similar to VSV, the incubation of splenic pDC subsets from WT mice with M-CoV for 14 h induced the highest levels of IFN-I in pDC-A, lower levels in pDC-B and no response in tDCs as measured by ELISA (Fig. 7e). We then cultured pDC subsets from the *Ifnb1*^EYFP^ reporter with M-CoV that encoded GFP (M-CoV-GFP) and analyzed EYFP and GFP expression at 8 h. The results were largely concordant with those from VSV-GFP, in that (1) the expression of *Ifnb1*^EYFP^ reporter and virus infection were mutually exclusive (Fig. 7f); (2) pDC-A and pDC-B harbored the same fraction of EYFP^+^ cells, but pDC-A yielded a higher number of these cells (Fig. 7g); (3) pDC-A also yielded a higher number of GFP^+^ infected cells (Fig. 7h); (4) tDCs failed to express the *Ifnb1*^EYFP^ reporter and showed the highest rate of M-CoV infection. Collectively, the pDC-A subset show the highest IFN-I-producing capacity but also high susceptibility to virus infection; they are also clearly different from tDCs, which become infected but fail to produce IFN-I.

To confirm the differential IFN-I response of pDC subsets in vivo, and to further distinguish them from tDCs, we used *Cx3cr1*^GFP^ mice to isolate and adoptively transfer GFP^−/lo^ pDC-A and pDC-B or GFP^+^ tDCs. To enable the analysis of IFN-I production by adoptively transferred pDCs, we used recipient mice with a DC-specific conditional knockout of TCF4 (Tcf4^cKO^: *Tcf4*^flox/flox^ *Cd11c*-Cre), which manifest a profound defect of pDC differentiation and function^7,10^. At 1 h after the transfer, mice were infected with M-CoV, and 2 d later, IFNα was measured in the spleen lysates by ELISA (Fig. 7i). The detection of IFNα was validated in the spleens of control WT mice that were infected with M-CoV 2 d earlier (Fig. 7j). The transfer of pDC-A resulted in detectable IFNα production, whereas pDC-B or tDCs yielded IFNα levels near the detection limit (Fig. 7k). Thus, the pDC-A subset are superior to pDC-B or tDCs in their IFN-I response to M-CoV in vivo.

### pDC heterogeneity is associated with differential survival

**Fig. 8:**
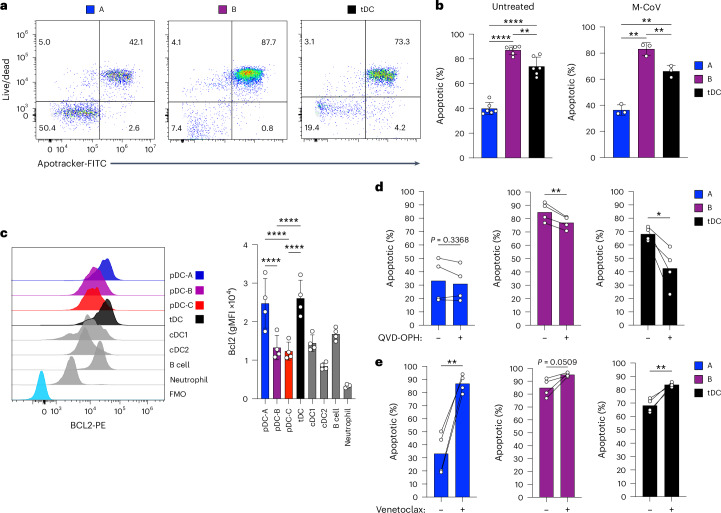
Differential survival of pDC subsets. **a**,**b**, The survival of pDC subsets in culture. Splenic pDC-A and pDC-B subsets or tDCs were sorted from WT mice, cultured with or without M-CoV for 8 h and stained for apoptotic/dead cells. Shown are representative staining profiles (**a**) and the fractions of dead cells in the cultures (**b**). Bars represent means ± s.d.; symbols represent biological replicates (cultures from individual mice) from three experiments (untreated) or two experiments (M-CoV). **c**, The expression of BCL2 in pDC subsets as determined by intracellular staining. Shown are representative histograms of BCL2 expression in the gated cell populations from B6 WT mouse spleen (left), and geometric mean fluorescence intensity (gMFI) of BCL2 expression (right). Bars represent means ± s.d.; symbols represent biological replicates (cultures from individual mice) from two experiments. FMO, fluorescence minus one. **d**,**e**, The role of BCL2 in the survival of pDC subsets in culture. pDC-A, pDC-B or tDCs were sorted from B6 WT mouse spleen, then cultured for 6 h with or without the caspase inhibitor Q-VD-OPH (**d**) or BCL2 inhibitor venetoclax (**e**), and cell death was determined the same as in **a** and **b**. Bars represent means ± s.d., symbols represent biological replicates (cultures from individual mice) from two experiments and lines connect paired samples from the same mouse. Statistical significance was analyzed using paired one-way ANOVA followed by Tukey’s test (**b**,**c**) or paired two-sided Student’s *t*-test (**d**,**e**). *P < 0.05; **P < 0.005; ***P < 0.0005; ****P < 0.00005. Statistics for comparisons with controls not shown: gray bars (**c**). Source data

The analysis of virus-induced IFN-I production (Fig. 7) showed that pDC-A and pDC-B harbor similar fractions of *Ifnb1*^+^ cells, yet the pDC-A subset yield higher *Ifnb1*^+^ cell numbers and produce more IFNα in culture. These results suggested that the enhanced IFN-I production by pDC-A may be related to their better survival following activation. When sorted pDC subsets were cultured for 8 h with or without M-CoV, >80% of pDC-B died (Fig. 8a,b), whereas pDC-A showed a >2-fold higher survival in both conditions. The survival of tDCs was intermediate between that of pDC-A and pDC-B (Fig. 8a,b), further highlighting the distinct characteristics of this cell population.

Searching for a possible molecular basis of the increased survival of pDC-A, we noticed their higher expression of *Bcl2* transcript by bulk RNA-seq (Supplementary Table 4). BCL2 is prominently expressed in pDCs compared with cDCs, and facilitates their survival when overexpressed^43^. Accordingly, intracellular staining showed higher levels of BCL2 protein in pDC-A compared with pDC-B or pDC-C (Fig. 8c). To test the role of BCL2 expression, we cultured pDC subsets with the caspase inhibitor Q-VD-OPH, and observed a significantly lower cell death in pDC-B and pDC-C, but not in pDC-A (Fig. 8d). Conversely, BCL2 inhibitor venetoclax increased the death of pDC-A in culture >2-fold, although it had no significant effect on the survival of pDC-B (Fig. 8e). The already high death rate of tDCs was also increased by venetoclax, consistent with their expression of BCL2 (Fig. 8c). These results reveal the downregulation of BCL2 and decreased survival of pDC-B/C subsets, which likely underlies their lower cytokine-producing capacity but also their lower ability to support virus replication.

## Discussion

Our single-cell analysis of peripheral pDC populations in naive mice revealed substantial transcriptional heterogeneity, with at least three distinct subpopulations defined by snRNA-seq and CITE-seq. Unlike the distinct populations observed in mouse^18–20,44^ or human^45^ pDCs following their activation, our observations reveal heterogeneity in quiescent pDCs. Notably, transcriptional heterogeneity was not reflected in chromatin accessibility, suggesting that it is generated during the relatively short lifespan of pDCs, following their lineage commitment. Moreover, pDCs were remarkably homogeneous in terms of their open chromatin profile, consistent with their likely derivation from a single source, which may be shared with cDCs as shown by lineage tracing in mice^32^ and humans^46^.

Consistent with its apparent post-commitment origin, the observed transcriptional (and the corresponding phenotypic) heterogeneity of pDCs was driven primarily by the ISGs and was fully dependent on cell-intrinsic IFNAR signaling. A similar ISG-driven heterogeneity was detected in human pDCs by tissue staining for MX1, and was also recently observed by scRNA-seq in quiescent pDCs from healthy individuals and patients with scleroderma^27^. Moreover, lineage tracing and adoptive transfers suggested that the three identified pDC subpopulations form a developmental progression, from the ‘IFN-I-naive’ pDC-A to the ‘IFN-I-primed’ pDC-B to the ‘IFN-I-experienced’ pDC-C. At least some relevant IFN-I may be produced by pDCs themselves, as evidenced by the history of *Ifnb1* expression in pDC-B and especially in pDC-C. This is consistent with the partial contribution of the TLR9 pathway to the generation of pDC heterogeneity, as TLR9-induced IFN-I production is a hallmark of pDCs. On the other hand, the pDC-B phenotype can be induced by exogenous IFN-I in vivo and by residual IFN-I in Flt3L culture, consistent with a cell-extrinsic IFN-I elicited through the cGAS/STING pathway. The commensal microbiome partially contributed to pDC heterogeneity, in line with the reported microbiome-driven tonic IFN-I production^35–37^. Additional stimuli may include endogenous retroviruses, which are known inducers of cGAS/STING as well as of endosomal TLRs including TLR3 (ref. ^47^). Finally, the local production and availability of IFN-I may shape tissue-specific heterogeneity and functionality of pDCs, the possibility to be explored in future studies.

Heterogeneous Sca1 expression on steady-state mouse pDCs has been described previously^23^ and correlated with IFN production. We used this and another IFN-I-inducible marker, CD69, to prospectively isolate three sequential stages of pDC differentiation, correlate them with single-cell data, and comprehensively characterize their function. Thus, ‘IFN-I-naive’ pDC-A showed superior ability to produce IFN-I and other cytokines; the majority subset of ‘IFN-I-primed’ pDC-B showed lower but still significant IFN-I production; and the pDC-C subset were profoundly dysfunctional. One caveat of these in vitro studies is the stimulation by soluble TLR ligands or viruses at high multiplicity of infection (MOI), whereas pDCs in vivo may be activated by virus-infected cells^3^. Notably, the stronger IFN-I response of pDC-A relative to pDC-B or tDC was also observed upon the transfer into virus-infected mice, although the precise quantitation of IFN-I production by pDC subsets in vivo remains to be achieved. These functional differences were associated with the downregulation of BCL2 and impaired survival of IFN-I-primed pDC-B. This is consistent both with the IFN-I-induced death of many pDCs following activation^48^ and with the dominant role of BCL2 in pDC survival^43^. The differential survival following virus-induced activation likely contributed to the superior IFN-I response by pDC-A during M-CoV infection in vivo.

The observed functional heterogeneity of pDCs may be relevant for several features of pDC-driven IFN-I responses. First, it reinforces the notion that only a minority of pDCs produce IFN-I, even in response to maximal stimulation. Second, it suggests that the reduction of tonic IFN-I signaling may facilitate cytokine responses by pDCs, as shown here for GF or STING-deficient mice. For instance, STING deficiency exacerbates autoimmunity in several models of systemic lupus erythematosus^49^, and our results provide a possible explanation for this paradoxical observation. Conversely, the impairment of pDC function by chronic IFN-I (as modeled here by adenovirus-based IFNα expression) may underlie the impaired functionality of pDCs in human patients with systemic lupus erythematosus^50^. Our results are also consistent with the functional impairment (‘exhaustion’) of pDCs in persistent virus infections, which facilitates superinfection by unrelated viruses^51,52^. Finally, a similar impairment of IFN-I production by pDCs has been described in tumors, where it may promote an immunosuppressive environment^53^. Our data suggest that these abnormalities may result in part from the elimination of the most functional pDC subset.

Why are the majority of peripheral pDCs driven by tonic IFN-I signaling into lower responsiveness and reduced survival? This may reflect an inevitable ‘side effect’ of pDC biology, including their unique IFN-I-producing capacity and high sensitivity to IFN-I (for example, due to high expression of IFNAR). Conversely, this heterogeneity may also represent a desirable feature, as suggested by its apparent evolutionary conservation. For instance, it may represent an additional layer of negative control; indeed, elevated IFN-I production by pDCs due to the lack of inhibitory receptors such as SiglecH^54^ or Ptprs^55^ causes inflammatory disease. Furthermore, the low survival of IFN-I-primed pDCs reduced not only their cytokine production, but also their infection by viruses including VSV and M-CoV. The latter feature, imparted by IFN-I, may underlie the resistance of pDCs to the majority of tested viruses. This notion is consistent with the increased susceptibility of IFNAR-deficient pDCs to mouse cytomegalovirus infection^56^. Furthermore, the increased survival of pDC-A may allow their differentiation into cDC-like cells^20^, which may present virus-derived antigens to T cells. Finally, given the cooperative nature of pDC response^15,57,58^, pDC-A may facilitate IFN-I production by pDC-B in vivo.

In conclusion, our single-cell analysis revealed phenotypic, transcriptional and functional heterogeneity of mature pDCs in the steady state. This heterogeneity is generated by tonic IFN-I derived from multiple cellular sources and sensing pathways, and results in the acquisition by the majority of pDCs of antiviral state at the expense of lower IFN-I-producing capacity. These observations shed light on multiple aspects of pDC biology, including the partial nature of IFN-I responses, the resistance to cell-intrinsic virus infection and the phenomenon of ‘exhaustion’ in chronic autoimmune and infectious diseases.

## Methods

Please see the Reporting Summary for details of mouse strains, antibodies and software used.

### Mice

Animal maintenance and experimentation were performed under the investigators’ protocols approved by the Institutional Animal Care and Use Committees of New York University Grossman School of Medicine (NYUGSoM) or of the University of California San Diego (UCSD) School of Medicine. WT C57BL/6 mice were obtained from Taconic and bred in-house. GF C57BL/6 mice were maintained in the GF facility at NYUGSoM. *Ifnar1*^−/−^ (B6.129S2-*Ifnar1*^tm1Agt^/Mmjax), *Sting1*^−*/*−^ (C57BL/6J-*Sting1*^gt^/J), *Cgas*^−/−^ (B6(C)-*Cgas*^tm1d(EUCOMM)Hmgu^/J) and OT-II (B6.Cg-Tg(TcraTcrb)425Cbn/J) mice were obtained from The Jackson Laboratory. *Unc93b1*^−/−^ (C57BL/6J-*Unc93b1*^*3d*^/Mmucd) mice were obtained from the Mutant Mouse Resource & Research Centers (MMRRC) repository. *Sting1*^−/−^ and *Tlr9*^−/−^ mice were intercrossed to obtain *Sting1*^−/−^*Tlr9*^−/−^ double-deficient mice. Reporter strains for *Mx1* (*Mx1*^GFP^, B6.Cg-*Mx1*^tm1.1Agsa^/J, ref. ^59^), *Ifnb1* (*Ifnb*^mob^, B6.129-*Ifnb1*^tm1Lky/J^, ref. ^17^) and *Cx3cr1* (*Cx3cr1*^eGFP^, B6.129P2(Cg)-*Cx3cr1*^tm1Litt/J^, ref. ^60^) were obtained from The Jackson Laboratory. The *Cx3cr1*^CreER^ *Rosa26*^LoxStopLox-Tom^ mice^32^, *hCD2*-Cre *Rosa26*^LoxStopLox-EYFP^ mice^10,32^, *Tcf4*^flox/flox^ *Cd11c*-Cre^+^ mice^7^ and *Tlr7*^−/−^*Tlr9*^−/−^ mice^61^ have been described. *Ifnb1*^tdTom-iCre^ (ref. ^33^) were crossed with *Rosa26*^LoxStopLox-EYFP^ (B6.Cg-*Gt(ROSA)26Sor*^*tm3(CAG-EYFP)Hze*^/J, The Jackson Laboratory) mice to obtain doubly-heterozygous *Ifnb1*^tdTom-iCre/+^ *Rosa26*^LoxStopLox-EYFP/+^ mice. For inducible lineage tracing in *Cx3cr1*^CreER^ *Rosa26*^LoxStopLox-Tom^ mice, mice were gavaged once with 0.1 ml of emulsion containing 5 mg of tamoxifen (Sigma-Aldrich) in sunflower seed oil, and euthanized 2–15 d later as indicated.

### BM chimeras

To generate reciprocal chimeras between WT 57BL/6 and *Sting1*^−/−^ mice, mice of either genotype were lethally irradiated and injected intravenously with 2 × 10^6^ total BM cells from donor mice of either genotype. To generate mixed BM chimeras, 10^6^ BM cells from *Ifnar1*^−/−^ or WT C57BL/6 mice were mixed with 10^6^ BM cells from CD45.1 congenic C57BL/6 mice (B6.SJL-*Ptprc*^*a*^*Pepc*^*b*^/BoyCrl, Charles River) and injected intravenously into lethally irradiated CD45.1 mice. Hematopoietic reconstitution was confirmed by flow cytometry analysis of peripheral blood.

### Adoptive transfers

To test the differentiation of pDC subsets in the steady state (Fig. 3g,h), pooled splenocytes from *Cx3cr1*^GFP^ CD45.1 mice were used to isolate GFP^−/lo^ pDC-A or pDC-B, or Cx3cr1^+^ tDCs. Cells (30,000 per recipient) were injected intravenously into WT B6 CD45.2 nonirradiated recipients, and splenic pDCs were analyzed 2 d later by flow cytometry. To test IFN-I production in vivo (Fig. 7i,j), cells were isolated as above and injected intravenously into *Tcf4*^flox/flox^ *Cd11c-*Cre^*+*^ mice, which were infected with 50 plaque-forming units of M-CoV (MHV A59, Bei Resources) intraperitoneally. At 2 d post transfer, recipient mice were euthanized, their spleens were homogenized and total protein concentration was measured using the BCA protein assay kit. IFNα levels were quantified from 10 μg of total protein using the high-sensitivity all-subtype IFNα ELISA kit.

### In vivo treatments

For in vivo IFNAR1 blockade experiments, mice were administered 0.2 mg of anti-IFNAR1 or isotype control antibody in PBS by intraperitoneal injection at the indicated time points. For chronic IFN-I exposure experiments, mice were injected intravenously with adenovirus vector encoding mouse IFNα5 (Ad-mIFNα5, Welgen)^62^ or an empty adenoviral vector (Welgen) at 1 × 10^10^ viral particles in PBS. For in vivo pDC stimulation, 5 μg of CpG-A was formulated with DOTAP and administered intravenously in 100 μl of sterile PBS. Peripheral blood was collected 6 h later by cheek bleed, and IFNα and IFNβ were measured in serum samples by ELISA using VeriKine-HS kits (PBL).

### In vitro DC development

To generate Flt3L, FLT3L-secreting clone of B16 melanoma^63^ was cultured in DMEM medium supplemented with 10% FBS, 1% l-glutamine, 1% sodium pyruvate, 1% nonessential amino acids (MEM-NEAA) and 1% penicillin/streptomycin at 37 °C in a humidified atmosphere at 5% CO_2_, and the supernatant was collected. Total BM cells were plated at 10^6^ cells per ml and cultured in complete DMEM supplemented with the 10% B16-Flt3L supernatant for the indicated length of time. Anti-IFNAR1 or isotype control antibodies were added at 10 μg ml^−1^ at the indicated time points.

### In vitro treatments

pDCs were seeded in 250 μl of complete DMEM per well in 96-well round-bottom plates at 2 × 10^4^ cells per well for FACS-sorted pDCs, and 1 × 10^6^ cells per well for DC-enriched splenocytes or total BM cells. Cells were stimulated with 1 μM CpG-A (ODN 2216), 5 μM CpG-B (ODN 1668) or 5 μg ml^−1^ ssRNA40. Supernatants were collected at 12–18 h, and analytes were measured by ELISA (Lumikine Xpress mIFNa 2.0 ELISA kit, InvivoGen) or bead array (Legendplex Mouse Anti-Virus Response Panel, BioLegend). Recombinant VSV expressing chicken OVA peptide SIINFEKL in frame with enhanced green fluorescent protein (VSV-GFP) was constructed as previously described^64^. It is based on the Indiana strain and is not known to have the M51R mutation of the matrix protein, and as such is capable of inhibiting the host gene expression. The resulting VSV with GFP was produced and titrated in BHK cells and added to cells at MOI of 10. M-CoV (MHV A59 expressing GFP^65^) was kindly provided by S. Weiss, and was used at MOI of 1. For experiments utilizing DC-enriched splenocytes or total BM cells (Figs. 5, 6 and 7a), absolute pDC number per well was calculated from flow cytometry analysis and used to normalize measured analyte concentrations across samples. For experiments in Figs. 7b–h and 8, pDC subsets and tDCs were sorted from *Ifnb*^mob^ (*Ifnb1*^EYFP^) mice, seeded at 2 × 10^5^ per ml, incubated with VSV-GFP or M-CoV-GFP and analyzed by spectral flow cytometry 8 h later. To measure IFN-I production, cells were cultured for 14–16 h, and IFNα was measured in the supernatants using the high-sensitivity all-subtype IFNα ELISA kit.

To measure cell survival, pDCs or tDCs from the spleens of WT mice were sorted as above, cultured for 6 h, stained with the Apotracker Green kit (BioLegend) and analyzed by flow cytometry. Venetoclax (SelleckChem) or Q-VD-Oph (5 μM, SelleckChem) or a vehicle (DMSO) was added as indicated.

To measure antigen presentation, T cells were purified from peripheral lymph nodes of OT-II mice using Mouse Pan T Cell Isolation Kit II (Miltenyi Biotec). In parallel, pDC subsets and control cDC2s were sorted from WT mice as described above. T cells and DCs (3 × 10^4^ each) were co-cultured in 0.25 ml of complete DMEM supplemented with 10 μg ml^−1^ OVA_323–339_ peptide or 0.1 mg ml^−1^ full-length OVA protein. Cells were collected at 64 h, and OVA-specific CD4^+^ T cells (SiglecH^−^TCRβ^+^CD4^+^TCR-Vβ-5.1/2^+^) were identified and enumerated by flow cytometry analysis.

### Primary cell preparation

Bones and lymphoid organs were dissected from euthanized mice. BM was flushed with cold complete DMEM (DMEM supplemented with 10% FBS, 1% l-glutamine, 1% sodium pyruvate, 1% MEM-NEAA, 1% penicillin/streptomycin, 55 μM 2-mercaptoethanol, 25 mM HEPES pH 7.4) and pressed through a 70-μm cell strainer to yield single-cell suspension. Spleen, lymph nodes and thymus were minced and pressed through a 70-μm cell strainer to yield single-cell suspension. Cells were treated with 1 × RBC lysis buffer (BioLegend) to remove red blood cells and washed with complete DMEM. For procedures necessitating pDC pre-enrichment, magnet-assisted cell sorting (MACS) depletion or BSA gradient was used, as indicated. For MACS depletion, post-RBC-lysis cells were stained for 15 min on ice in MACS buffer (PBS supplemented with 0.5% BSA and 2 mM EDTA) with biotinylated antibodies for the following markers: CD3e, TCRβ, CD90.2 (Thy1.2), NK1.1, CD11b, F4/80, Ly6G, CD24, CD138, IgM and IgD. Cells were washed in MACS buffer, then incubated for 15 min on ice in MACS buffer with Streptavidin Microbeads (Miltenyi Biotec) at 1/4th the manufacturer’s recommended concentration. Cells were washed in MACS buffer, resuspended in MACS buffer, run over MACS LS columns (Miltenyi Biotec) and flow-through collected as enriched samples to be used for downstream applications.

For DC enrichment by BSA gradient, a 30% solution of BSA was prepared or purchased (MP Biomedicals). Cells were taken immediately from the initial single-cell suspension (without RBC lysis), transferred to a 2-ml microcentrifuge tube, centrifuged for 5 min at 500*g* and 4 °C and resuspended in 1 ml of 30% BSA. A layer of 0.8 ml of cold PBS was then added carefully, so as to prevent mixing of layers. Samples were centrifuged for 15 min at 9,400*g* and 4 °C. Upper PBS layer, DC-enriched interphase and half of the BSA layer were all collected, while carefully avoiding the pellet. This DC-enriched fraction was then washed in complete DMEM, and used for downstream applications.

For experiments in Figs. 7 and 8, mouse spleens were first digested with 400 U ml^−1^ Collagenase D and 50 μg ml^−1^ DNase I for 25 min at 37 °C. Subsequently, 10 mM EDTA was added, and cells were incubated for an additional 5 min at 37 °C. After red blood cell lysis, splenocytes were washed and filtered through a 70-μm cell strainer to obtain a single-cell suspension.

### Flow cytometry

Cells were stained with fluorophore-conjugated antibodies for 20–60 min in FACS buffer (PBS supplemented with 1% FBS, 1% BSA and 5 mM EDTA) on ice. Fixable viability dye (eBioscience) or DAPI was used to identify dead cells. For analysis, samples were acquired on an Attune NxT (Thermo Fisher Scientific) using Attune NxT software and further analyzed with FlowJo software (Tree Star).

Cell sorting was performed using a 100-μm nozzle on a BD FACSAria IIu SORP flow sorter (Becton Dickinson) at the Cytometer & Cell Sorting Laboratory at New York University School of Medicine. For CITE-seq, splenocytes were isolated from B6 WT mice and pre-enriched by MACS depletion. Mature pDCs were sorted as IgM^−^IgD^−^TCRb^−^B220^+^Ly6C^+^. For bulk RNA-seq, splenocytes were isolated from B6 WT and *Ifnar1*^−/−^ mice and pre-enriched by MACS depletion. Mature pDCs were sorted as CD11c^int^PDCA1^+^, and further subdivided by expression of Sca1 and CD69. For functional analyses, splenocytes were isolated from B6 WT mice and pre-enriched by MACS depletion or BSA gradient. Mature pDCs were sorted as CD11c^int^PDCA1^+^Cx3cr1^−^, and further subdivided by expression of Sca1 and CD69. For control samples, CD11c non-pDCs were sorted as CD11c^hi^PDCA1^−^ and cDC2 were sorted as CD11c^hi^PDCA1^−^CD11b^+^. For multiome analysis, splenocytes were isolated from B6 WT mice and pre-enriched by BSA gradient. Mature pDCs were sorted as CD11c^int^PDCA1^+^Cx3cr1^−^CD11b^−^.

For experiments shown in Figs. 4g–j, 7 and 8, mouse spleen cell suspensions were incubated with anti-CD135-biotin for 30 min at 4 °C, washed and then incubated with Ultra-Pure anti-Biotin microbeads (Miltenyi) for 45 min at 4 °C. CD135^+^ cells were enriched using LS magnetic columns, and pDC subpopulations and tDCs were sorted using a FACS Fusion (BD Biosciences). In brief, from a single, live-cell population, lineage-positive cells (CD3, CD19, NK1.1 and Ly6G) were excluded. pDCs were gated as XCR1^−^CD11b^−^CX3CR1^−^Ly6C^+/low^SiglecH^+^. From this gate, pDC-A subset was sorted as CD69^−^SCA1^−^; pDC-B as CD69^−^SCA1^+^; and pDC-C as CD69^+^SCA1^+^. tDCs were gated as Lin^−^XCR1^−^CD11b^−^CX3CR1^+^CD11c^+^SiglecH^−/int^Ly6C^low/high^ as previously described^10^.

### Infinity Flow

The general Infinity Flow approach has been described^66^. Splenocytes from hCD2-EYFP mice were isolated and stained for 45 min at 4 °C with 13 backbone markers. The backbone-labeled cells were split and stained with 39 different PE-conjugated antibodies for 45 min at 4 °C. All samples were acquired on the 5-laser Cytek Aurora flow cytometer (Cytek). After data collection, FCS files were individually examined in FlowJo for compensation adjustment and quality control, gated on the Live^+^Lin (CD3, CD19, Ly6G, NK1.1)^−^CD11c^+^ cells for each sample and were exported into a new FCS file. All exported FCS files were used as input for the Infinity Flow prediction pipeline using the Infinity Flow R package^66^. All the Infinity Flow output files were concatenated into one FCS file for FlowSOM analysis and UMAP generation in FlowJo.

### Single-cell multiome

Splenic mature pDCs were sorted, and nuclei were isolated according to a 10x Genomics protocol (CG000365, revision C) with the lysis time of 4 min. Nuclei were then loaded onto the 10x Genomics Chromium Controller, and single-nucleus ATAC-seq and RNA-seq library preparation and sequencing were performed using the Chromium Single Cell Multiome ATAC + Gene Expression kit (10x Genomics) according to manufacturer’s instructions at the Genomics Core facility, NYUGSoM.

### Single-cell multiome data analysis

The 10x Genomics CellRanger arc v.2.0.0 pipeline was used to process .fastq files corresponding to the RNA-seq transcripts and ATAC-seq reads into barcode-filtered counts tables, and call peaks in the latter. The Seurat and Signac packages^67,68^ were used for the import, normalization, scaling and downstream processes of clustering and visualization. For the chromatin component, the following quality control filters were applied: 20,000 > peak region fragments > 3,000; percentage of reads in called peaks > 15%; mm10 blacklist ratio reads in peaks < 10%; nucleosome signal (ratio of nucleosome-bound to nucleosome-free fragments) < 4; transcription start site enrichment (ratio of the number of reads mapping near to transcription start sites across the genome to the number of reads mapping to distal flanking regions) > 2. The following criteria were applied for the RNA component: 25,000 > number of reads per cell > 1,000; percentage of reads per cell mapping to mitochondrial genome < 20%. The transcriptome was normalized using the Seurat Single Cell Transform method, and the ATAC-seq normalized using latent semantic indexing. UMAPs generated for each assay were constructed using 50 principal components (PCs) (with the first latent semantic indexing component in the ATAC-seq skipped as it exhibits robust correlation to sequencing depth). The SingleR correlation-based cellular-level phenotyping tool was used in conjunction with the Immgen database to generate cell type labels for the transcriptome and served as the basis for the elimination of non-DC contaminants.

For pDC and tDC module score analysis, the following gene signatures for pDCs and tDCs were used as inputs to the Seurat *AddModuleScore* function: (1) pDC: ‘Tcf4’, ‘Siglech’, ‘Runx2’, ‘Bcl11a’, ‘Bst2’, ‘Klra17’, ‘Irf7’; (2) tDC: ‘Cx3cr1’, ‘Irf4’, ‘Cd14’, ‘Csf1r’, ‘Ms4a6c’, ‘Tmem176b’, ‘Slamf7’, ‘Klf4’, ‘Spi1’, ‘Fam46a’, ‘Zbtb46’. Background-normalized expression profiles for each signature were then generated and projected on the UMAP of the transcriptome analysis. For cluster correlation analyses used in cluster resolution optimization, the cluster marker sets (marker genes for RNA component) were computed using the Seurat FindAllMarkers function with default settings at resolution 0.8 using the Louvain algorithm, with multilevel refinement via Seurat’s FindClusters function on the normalized RNA assay. Next, an *m* × *n* matrix for *n* clusters and *m* total unique markers was constructed across all clusters; each entry *M*_*ij*_ corresponding to the average expression of marker *i* across all cells in cluster *j*. The Pearson correlation between all clusters was then computed. The subsequent correlation matrix was passed as input to the ComplexHeatmap R package’s Heatmap function. The same analysis was repeated for various clustering resolutions on the ATAC-seq component using the marker set of differentially accessible regions (DARs).

To define TF motifs, DARs between ATAC-seq clusters were identified using the Seurat FindAllMarkers function. A strict threshold was applied retaining only those peaks with average log_2_ fold-change in the 1-v-all comparison > 1, as well as an adjusted *P* < 0.1. The FindMotifs Signac function was then independently applied to each cluster’s resulting DARs, subsetting by fold-change > 1 and adjusted *P* < 0.1 on the resulting motifs. To define the cluster-enriched motifs, a custom web-scraper was built to query the JASPAR online motif database and pull motif family fields by motif ID returned by the FindMotifs function. Finally, for each cluster, faceted plots were generated using ggplot2 R package, with motifs sorted in descending order by −log_10_ of adjusted enrichment *P* value, grouped by TF family.

### CITE-seq

Antibodies used in CITE-seq were purchased as purified IgG and conjugated using iEDDA click chemistry to barcode oligos as described^69^. CITE-seq and cell hashing were performed as described previously^70,71^. After MACS depletion, before FACS sorting, each sample was stained with a pool of ADT- and sample-specific hashtagging oligonucleotide-conjugated antibodies. After staining and sorting, cells were run through the standard 10x Chromium (v3) protocol, with subsequent complementary DNA amplification, cleanup and construction of transcriptome, ADT and hashtagging oligonucleotide libraries performed as described^32^. Libraries were pooled and sequenced on a 100 cycle Novaseq S1 flowcell.

### CITE-seq data analysis

For quality control, we confirmed the integrity of the cDNA, quality of the libraries, number of cells sequenced and mean number of reads per cell. RNA library sequenced reads were aligned to *Mus musculus* reference genome (mm10/GRCm38) using CellRanger (10x Genomics). Unique molecular identifier correction was performed to handle PCR duplications. Quality control was then performed by calculating the number of genes, unique molecular identifiers and the proportion of mitochondrial genes for each cell using iCellR R package^72^. Cells were filtered out if they did not meet the following quality control thresholds: (1) ratio of reads mapping to mitochondrial/nuclear < 0.1; (2) minimum number of genes > 500; (3) maximum number of genes < 5,000. Then the gene–cell matrix was normalized based on ranked geometric library size factor using iCellR. We used only the top ranked genes (top 500 genes sorted by base mean) to reduce the effect of dropouts (nonzero events counted as zero) in normalization by considering only the highly expressed genes. A general statistical test was then performed to calculate gene dispersion, base mean and cell coverage to build a gene model for principal component analysis (PCA). Genes with high coverage (top 500) and high dispersion (dispersion > 1.5) were chosen and PCA was performed. Then the clustering was performed (iCellR options; clust.method = ‘kmeans’, dist.method = ‘euclidean’, index.method = ‘silhouette’) on the PCs with high standard deviation (typically top 7–10 PCs). UMAP^73^ was also performed on the top PCs.

Marker genes for each cluster were determined based on fold-change and adjusted *P* value (*t*-test) and average gene expression for each cluster was calculated using iCellR. Marker genes were visualized on heatmaps, bar plots and box plots for each cluster. Marker genes were used to determine the predominant cell types composing each cluster by manual curation and correlation to ImmGen datasets^74^, which was used to exclude non-pDC cellular contaminants.

RNA velocity analysis was performed as described^75^.

### RNA-seq data analysis

Sequencing results were demultiplexed and converted to FASTQ format using Illumina bcl2fastq software. The FASTQ files were further processed using Seq-N-Slide pipeline. The sequencing reads were adapter and quality trimmed with Trimmomatic and then aligned to the mouse genome (build mm10/GRCm38) using the splice-aware STAR aligner. The FeatureCounts program was utilized to generate counts for each gene based on the number of aligned reads that overlap its exons. These counts were then normalized and used to test for differential expression using negative binomial generalized linear models implemented by the DESeq2 R package.

The results (Supplementary Table 4) suggest that a minor fraction of Cx3cr1^+^ tDCs (which are Sca1^−^ and were not gated out in this experiment) was likely present in the pDC-A subset as evidenced by tDC-specific genes. Similarly, a minor fraction of natural killer cells may have been present in the pDC-C subset as evidenced by natural killer cell-specific genes. However, neither tDC- nor natural killer cell-derived gene sets affected the analysis of the ISG signature in this dataset.

### Analysis of previously published datasets

For a previously published mouse pDC dataset (GSE114313)^30^, gene symbols were converted from Ensembl IDs using the org.Mm.eg.db Bioconductor package. Using Seurat^67^, the dataset was filtered to remove cells that did not meet these thresholds: minimum genes per cell > 1,000, maximum genes per cell < 4,000, percentage of mitochondrial RNA < 10. The log normalization and linear scaling were performed, and variable features were identified by variance stabilizing transformation followed by PCA. Leiden clustering^76^ was used with 50 PCs and 15 nearest neighbors, followed by further dimensional reduction using UMAP. The expression of *Ccr9* and *Klra17* was used to identify mature pDCs, and only mature splenic pDCs were selected for further analysis. Clustering was repeated for each of the three samples of splenic pDCs, and a cluster of contaminating tDCs was identified and excluded. Marker genes of resulting clusters were identified using a Wilcoxon rank sum test, and iCellR was used for KNetL, with the KNetL zoom parameter set to 200.

For a previously published human pDC dataset (GSE189120), the Seurat workflow was performed as described above but with an added round of canonical correlation analysis to remove batch effects. This batch correction removed the heterogeneity within resting pDCs that was described in the original publication^26^. Similarly, tDCs were removed based on previously published signatures, and clustering analysis and KNetL visualization were performed with the zoom parameter at 100.

### Human tissue staining

Human lymph nodes were obtained from brain-dead organ donors through an approved protocol and material transfer agreement with LiveOnNY, the local organ procurement organization for the New York metropolitan area, as previously described^77,78^. Tissues for this study were obtained from donors with no history of cancer, hepatitis B and C, or human immunodeficiency virus. Lymph nodes were formalin-fixed, paraffin-embedded, sectioned and mounted on coverslips coated with Vectabond tissue section adhesive (Vector Laboratories). Staining was performed using the CODEX (CO-Detection by indEXing) multiplexed imaging platform^79^. Purified antibodies against TCF4, IRF8 and MX1 were tagged with fluorophore-conjugated oligonucleotides using the Antibody Conjugation Kit (Akoya Biosciences). The tissue was then stained using the Staining Kit for Phenocycler (Akoya Biosciences), with the staining time adjusted to overnight at 4 °C. Imaging was performed on the Keyence BZ-X700 automated fluorescent microscope connected to the CODEX microfluidics system (Akoya Biosciences). The raw images were converted into a qptiff file using the CODEX processing software and visualized using HALO software (Indicalab).

### Quantification and statistical analysis

Comparison of different experimental groups was done using Prism software (GraphPad). Unless indicated otherwise, statistical significance was analyzed using one-way analysis of variance (ANOVA) followed by Tukey’s test. The normal distribution of values was tested using quantile–quantile plots.

### Reporting summary

Further information on research design is available in the Nature Portfolio Reporting Summary linked to this article.

## Online content

Any methods, additional references, Nature Portfolio reporting summaries, source data, extended data, supplementary information, acknowledgements, peer review information; details of author contributions and competing interests; and statements of data and code availability are available at 10.1038/s41590-025-02279-4.

## Supplementary information

Reporting Summary

Supplementary Table 1 Marker genes of cell clusters in the single-nucleus RNA-seq of mouse pDCs.

Supplementary Table 2 Marker peaks of cell clusters in the single-nucleus ATAC-seq of mouse pDCs.

Supplementary Table 3 Marker genes of cell clusters in the CITE-seq of mouse pDCs.

Supplementary Table 4 Gene expression in prospectively isolated subsets of mouse pDCs.

## Source data

Source Data for Figs. 1–8 and Extended Data Figs. 1–7 Statistical source data.

Source Data Fig. 2 Raw image files (JPEG) for Fig. 2g,h.

## Data Availability

All sequencing data have been deposited in the NCBI Gene Expression Omnibus database under accession number GSE252191. All other data are presented in the paper or are available from the corresponding authors upon request. Source data are provided with this paper.
