# Supplementary material for: Tonic type I interferon signaling optimizes the antiviral function of plasmacytoid dendritic cells
Source: Nat Immunol. 2025 Oct 14;26(11):1946–61. doi: 10.1038/s41590-025-02279-4 (PMC12662804; doi:10.1038/s41590-025-02279-4)
Supplement: Supplementary file 1 — Reporting Summary [file 41590_2025_2279_MOESM1_ESM.pdf]

nature portfolio

Corresponding author(s): Boris Reizis

Last updated by author(s): Aug 4, 2025

Reporting Summary

Nature Portfolio wishes to improve the reproducibility of the work that we publish. This form provides structure for consistency and transparency in reporting. For further information on Nature Portfolio policies, see our Editorial Policies and the Editorial Policy Checklist.

Statistics

For all statistical analyses, confirm that the following items are present in the figure legend, table legend, main text, or Methods section.

n/a	Confirmed	
☐	☒	The exact sample size (*n*) for each experimental group/condition, given as a discrete number and unit of measurement
☒	☐	A statement on whether measurements were taken from distinct samples or whether the same sample was measured repeatedly
☐	☒	The statistical test(s) used AND whether they are one- or two-sided<br>*Only common tests should be described solely by name; describe more complex techniques in the Methods section.*
☐	☒	A description of all covariates tested
☐	☒	A description of any assumptions or corrections, such as tests of normality and adjustment for multiple comparisons
☐	☒	A full description of the statistical parameters including central tendency (e.g. means) or other basic estimates (e.g. regression coefficient) AND variation (e.g. standard deviation) or associated estimates of uncertainty (e.g. confidence intervals)
☐	☒	For null hypothesis testing, the test statistic (e.g. *F*, *t*, *r*) with confidence intervals, effect sizes, degrees of freedom and *P* value noted<br>*Give P values as exact values whenever suitable.*
☒	☐	For Bayesian analysis, information on the choice of priors and Markov chain Monte Carlo settings
☒	☐	For hierarchical and complex designs, identification of the appropriate level for tests and full reporting of outcomes
☒	☐	Estimates of effect sizes (e.g. Cohen's *d*, Pearson's *r*), indicating how they were calculated

Our web collection on statistics for biologists contains articles on many of the points above.

Software and code

Policy information about availability of computer code

Data collection	CODEX® Processor 1.8.2.13 || Akoya Biosciences || https://help.codex.bio/codex/processor/installation/download-and-install
Data analysis	FlowJo v10.8.1 || FlowJo, LLC || https://www.flowjo.com/<br>Prism 9 || GraphPad || https://www.graphpad.com/<br>samtools (v.1.3.1) || 19505943 || http://samtools.sourceforge.net/<br>GenomicAlignments (v.1.10.1) || 23950696 || https://bioconductor.org/packages/release/bioc/html/GenomicAlignments.html<br>Bedtools2 (v2.26.0) || 20110278 || https://github.com/arq5x/bedtools2<br>Bowtie2 (v2.2.9) || 22388286 || http://bowtie-bio.sourceforge.net/bowtie2/index.shtml<br>UCSC mm10 known Gene Annotation Database (v.3.4) || 26590259 || https://bioconductor.org/packages/release/data/annotation/html/TxDb.Mmusculus.UCSC.mm10.knownGene.html<br>BLAT || 20639541 || https://genome.ucsc.edu/cgi-bin/hgBlat<br>Cutadapt (v 1.18) || https://doi.org/10.14806/ej.17.1.200 || https://cutadapt.readthedocs.io/en/stable/<br>iCellR || https://doi.org/10.1101/2020.03.31.019109 || https://github.com/rezakj/iCellR<br>KNetL || https://doi.org/10.1101/2020.05.05.078550 || https://github.com/rezakj/iCellR<br>Seurat v3.0 || 31178118 || https://satijalab.org/seurat/<br>Seq-N-Slide || Dr. Igor Dolgalev || https://igordot.github.io/sns/<br>bcl2fastq || Illumina || https://support.illumina.com/downloads/bcl2fastq_conversion_software_184.html<br>Trimmomatic || 24695404 || http://www.usadellab.org/cms/index.php?page=trimmomatic<br>STAR aligner || 23104886 || http://code.google.com/p/rna-star/<br>featureCounts || 24227677 || https://bioconductor.org/packages/3.18/bioc/html/Rsubread.html<br>DESeq2 || 25516281 || http://www.bioconductor.org/packages/release/bioc/html/DESeq2.html

HALO® v3.6 || Indica Labs || https://indicalab.com/

For manuscripts utilizing custom algorithms or software that are central to the research but not yet described in published literature, software must be made available to editors and reviewers. We strongly encourage code deposition in a community repository (e.g. GitHub). See the Nature Portfolio guidelines for submitting code & software for further information.

Data

Policy information about availability of data

All manuscripts must include a data availability statement. This statement should provide the following information, where applicable:

- Accession codes, unique identifiers, or web links for publicly available datasets
- A description of any restrictions on data availability
- For clinical datasets or third party data, please ensure that the statement adheres to our policy

All sequencing data have been deposited in the NCBI Gene Expression Omnibus database under the accession number GSE252191

Research involving human participants, their data, or biological material

Policy information about studies with human participants or human data. See also policy information about sex, gender (identity/presentation), and sexual orientation and race, ethnicity and racism.

Reporting on sex and gender	*Use the terms sex (biological attribute) and gender (shaped by social and cultural circumstances) carefully in order to avoid confusing both terms. Indicate if findings apply to only one sex or gender; describe whether sex and gender were considered in study design; whether sex and/or gender was determined based on self-reporting or assigned and methods used. Provide in the source data disaggregated sex and gender data, where this information has been collected, and if consent has been obtained for sharing of individual-level data; provide overall numbers in this Reporting Summary. Please state if this information has not been collected. Report sex- and gender-based analyses where performed, justify reasons for lack of sex- and gender-based analysis.*
Reporting on race, ethnicity, or other socially relevant groupings	*Please specify the socially constructed or socially relevant categorization variable(s) used in your manuscript and explain why they were used. Please note that such variables should not be used as proxies for other socially constructed/relevant variables (for example, race or ethnicity should not be used as a proxy for socioeconomic status). Provide clear definitions of the relevant terms used, how they were provided (by the participants/respondents, the researchers, or third parties), and the method(s) used to classify people into the different categories (e.g. self-report, census or administrative data, social media data, etc.) Please provide details about how you controlled for confounding variables in your analyses.*
Population characteristics	*Describe the covariate-relevant population characteristics of the human research participants (e.g. age, genotypic information, past and current diagnosis and treatment categories). If you filled out the behavioural & social sciences study design questions and have nothing to add here, write "See above."*
Recruitment	*Describe how participants were recruited. Outline any potential self-selection bias or other biases that may be present and how these are likely to impact results.*
Ethics oversight	It has been determined by the Institutional Review Board of Columbia University that the use of tissues from deceased organ donors does not constitute human subjects research.

Note that full information on the approval of the study protocol must also be provided in the manuscript.

Field-specific reporting

Please select the one below that is the best fit for your research. If you are not sure, read the appropriate sections before making your selection.

☒ Life sciences ☐ Behavioural & social sciences ☐ Ecological, evolutionary & environmental sciences

For a reference copy of the document with all sections, see nature.com/documents/nr-reporting-summary-flat.pdf

Life sciences study design

All studies must disclose on these points even when the disclosure is negative.

Sample size	Sample sizes were not determined a priori
Data exclusions	No data were deliberately excluded
Replication	Data on replication and summary statistics are provided in figure legends
Randomization	In experiments when the same animals were given different treatments (e.g. Fig. 6), the allocation into experimental groups was random.
Blinding	Blinding was not used because experimental setup and readout were performed by the same person(s)

Reporting for specific materials, systems and methods

We require information from authors about some types of materials, experimental systems and methods used in many studies. Here, indicate whether each material, system or method listed is relevant to your study. If you are not sure if a list item applies to your research, read the appropriate section before selecting a response.

Materials & experimental systems

n/a	Involved in the study
☐	☒ Antibodies
☐	☒ Eukaryotic cell lines
☒	☐ Palaeontology and archaeology
☐	☒ Animals and other organisms
☒	☐ Clinical data
☒	☐ Dual use research of concern
☒	☐ Plants

Methods

n/a	Involved in the study
☒	☐ ChIP-seq
☐	☒ Flow cytometry
☒	☐ MRI-based neuroimaging

Antibodies

Antibodies used

Flow cytometry
Figures 1-2, 3D-J, 4-6, 7A:
Anti-mouse IFNAR1 – PE (clone MAR1-5A3) || BioLegend || Cat# 127312
Anti-mouse Ly6A/E (Sca1) – PerCP-Cy5.5 (clone D7), eBioscience || Thermo Fisher Scientific || Cat# 45-5981-82
Anti-mouse CD69 – PE-Cy7 (clone H1.2F3), eBioscience || Thermo Fisher Scientific || Cat# 25-0691-82
Anti-mouse CD317 (Bst2, PDCA1) – APC (clone 927) || BioLegend || Cat# 127016
Anti-mouse CD317 (Bst2, PDCA1) – BV605 (clone 927) || BioLegend || Cat# 127025
Anti-mouse CD11c – eFluor450 (clone N418), eBioscience || Thermo Fisher Scientific || Cat# 48-0114-82
Anti-mouse CD11c – FITC (clone N418), eBioscience || Thermo Fisher Scientific || Cat# 11-0114-85
Anti-mouse CD45R (B220) – BV605 (clone RA3-6B2) || BioLegend || Cat# 103244
Anti-mouse CD45R (B220) – Pacific Blue (clone RA3-6B2) || BioLegend || Cat# 103227
Anti-mouse SiglecH – BV711 (clone 440c) || BD Biosciences || Cat# 747671
Anti-mouse SiglecH – PE (clone 440c), eBioscience || Thermo Fisher Scientific || Cat# 12-0333-82
Anti-mouse CD199 (CCR9) – FITC (clone CW 1.2), eBioscience || Thermo Fisher Scientific || Cat# 11-1991-82
Anti-mouse MHC Class II (I-A/I-E) – AF700 (clone M5/114.15.2) || BioLegend || Cat# 107622
Anti-mouse Cx3cr1 – APC-Cy7 (clone SA011F11) || BioLegend || Cat# 149048
Anti-mouse CD11b – APC-eFluor780 (clone M1/70), eBioscience || Thermo Fisher Scientific || Cat# 47-0112-82
Anti-mouse CD45 – APC (clone 30-F11) || BioLegend || Cat# 103112
Anti-mouse CD45.1 – FITC (clone A20), eBioscience || Thermo Fisher Scientific || Cat# 11-0453-82
Anti-mouse CD45.2 – PE (clone 104), eBioscience || Thermo Fisher Scientific || Cat# 12-0454-82
Anti-mouse CD44 – BV605 (clone IM7) || BD Biosciences || Cat# 563058
Anti-mouse CD4 – BV711 (clone RM4-5) || BioLegend || Cat# 100550
Anti-mouse CD25 – PerCP-Cy5.5 (clone PC61) || BioLegend || Cat# 102029
Anti-mouse TCRb – PECy7 (clone H57-597) || BioLegend || Cat# 109222
Anti-mouse TCR-Vb5.1/2 – APC (clone MR9-4) || BioLegend || Cat# 139506
Anti-mouse Ly6C – APC (clone HK1.4), eBioscience || Thermo Fisher Scientific || Cat# 17-5932-82
Anti-mouse CD62L (L-Selectin) – APC-eFluor780 (clone MEL-14), eBioscience || Thermo Fisher Scientific || Cat# 47-0621-82
Anti-mouse CD3ε – Biotin (clone 145-2C11) || BioLegend || Cat# 100304
Anti-mouse TCRb – Biotin (clone H57-597), eBioscience || Thermo Fisher Scientific || Cat# 13-5961-82
Anti-mouse CD90.2 (Thy1.2) – Biotin (clone 53-2.1) || BioLegend || Cat# 140314
Anti-mouse NK1.1 – Biotin (clone PK136), eBioscience || Thermo Fisher Scientific || Cat# 13-5941-85
Anti-mouse CD11b – Biotin (clone M1/70), eBioscience || Thermo Fisher Scientific || Cat# 13-0112-85
Anti-mouse F4/80 – Biotin (clone BM8), eBioscience || Thermo Fisher Scientific || Cat# 13-4801-85
Anti-mouse Ly6G – Biotin (clone 1A8) || BioLegend || Cat# 127604
Anti-mouse CD24 – Biotin (clone M1/69), eBioscience || Thermo Fisher Scientific || Cat# 13-0242-85
Anti-mouse CD138 (Syndecan-1) – Biotin (clone 281-2) || BioLegend || Cat# 142512
Anti-mouse IgM – Biotin (clone II/41), eBioscience || Thermo Fisher Scientific || Cat# 13-5790-85
Anti-mouse IgD – Biotin (clone 11-26c), eBioscience || Thermo Fisher Scientific || Cat# 13-5993-85
TruStain FcX anti-mouse CD16/32 antibody || BioLegend || Cat# 101320
Fixable Viability Dye (FVD) eFluor 506 || eBioscience || Cat# 65-0866-14
DAPI || Sigma-Aldrich || Cat# D8417

Figures 3K-M, 7B-J, 8:
Anti-mouse SiglecH PE || BD Biosciences || Cat# 552126
Anti-mouse XCR1 PercPCy5.5 || BioLegend || Cat# 148208
Anti-mouse CX3CR1 PeCy7 || BioLegend || Cat# 149016
Anti-mouse Sca-1 BV421 || BioLegend || Cat# 108127
Anti-mouse Ly6C BV510 || BioLegend || Cat# 128033
Anti-mouse CD11b BV785 || BioLegend || Cat# 101243

Anti-mouse CD69 APC || BioLegend || Cat# 104514
Anti-mouse CD3 AF700 || BioLegend || Cat# 100216
Anti-mouse CD19 AF700 || eBioscience || Cat# 48-0193-82
Anti-mouse NK1.1 AF700 || BioLegend || Cat# 108730
Anti-mouse Ly6G AF700 || BioLegend || Cat# 127622
Anti-mouse CD24 APC-eFluor 780 || eBioscience || Cat# 47-0242-82
Anti-mouse CD11c BUV737 || BD Biosciences || Cat# 612797
Anti-mouse CD172a || BD Biosciences || Cat# 740282
LIVE/DEAD™ Fixable Blue || Invitrogen || Cat# L23105

Figure 3A-C (Infinity Flow):
Backbone
Anti-mouse XCR1 – PerCP-Cy5.5 (clone ZET) || BioLegend || Cat# 148207
Anti-mouse ESAM – BV421 (clone 1G8) || BD Biosciences || Cat# 752436
Anti-mouse CD3ε – eFluor450 (clone 17A2), eBioscience || Thermo Fisher Scientific || Cat# 48-0032-82
Anti-mouse CD19 – eFluor450 (eBio1D3 (1D3)), eBioscience || Thermo Fisher Scientific || Cat# 48-0193-82
Anti-mouse NK1.1 – eFluor450 (clone PK136), eBioscience || Thermo Fisher Scientific || Cat# 48-5941-82
Anti-mouse Ly6G – eFluor450 (clone 1A8-Ly6g), eBioscience || Thermo Fisher Scientific || Cat# 48-9668-82
Anti-mouse MHC Class II (I-A/I-E) – BV510 (clone M5/114.15.2) || BioLegend || Cat# 107635
Anti-mouse Ly-6C – BV570 (clone HK1.4) || BioLegend || Cat# 128029
Anti-mouse CD11b – BV650 (clone M1/70) || BioLegend || Cat# 101239
Anti-mouse CD135 – Biotin (clone A2F10) || BioLegend || Cat# 135307
Streptavidin – BV785 || BioLegend || Cat# 405249
Anti-mouse SiglecH – APC (clone 551) || BioLegend || Cat# 129611
Anti-mouse CD43 – AF700 (clone S11) || BioLegend || Cat# 143213
Anti-mouse CD172a (SIRPα) – APC/Fire™ 750 (clone P84) || BioLegend || Cat# 144029
Anti-mouse Cx3cr1 – PE/Cyanine7 (clone SA011F11) || BioLegend || Cat# 149015
Anti-mouse CD24 – BUV 395 (clone M1/69) || BD Biosciences || Cat# 744471
Anti-mouse CD11c – BUV 737 (clone HL3) || BD Biosciences || Cat# 612796
PE conjugates:
Anti-mouse B220 – PE (clone RA3-6B2) || BioLegend || Cat# 103207
Anti-mouse CD192 (CCR2) – PE (clone SA203G11) || BioLegend || Cat# 150609
Anti-mouse CD197 (CCR7) – PE (clone 4B12) || BioLegend || Cat# 120105
Anti-mouse CD199 (CCR9) – PE (clone CW-1.2) || BioLegend || Cat# 128709
Anti-mouse CD103 – PE (clone 2E7), eBioscience || Thermo Fisher Scientific || Cat# 12-1031-82
Anti-mouse CD115 (CSF-1R) – PE (clone AFS98) || BioLegend || Cat# 135505
Anti-mouse CD117 (c-Kit) – PE (clone 2B8) || BioLegend || Cat# 105807
Anti-mouse CD127 – PE (clone A7R34), eBioscience || Thermo Fisher Scientific || Cat# 12-1271-82
Anti-mouse CD14 – PE (clone Sa2-8), eBioscience || Thermo Fisher Scientific || Cat# 12-0141-82
Anti-mouse CD169 (Siglec-1) – PE (clone 3D6.112) || BioLegend || Cat# 142403
Anti-mouse CD16/32 – PE (clone s17011e) || BioLegend || Cat# 156605
Anti-mouse CD200R (OX2R) – PE (clone OX-110) || BioLegend || Cat# 123907
Anti-mouse CD205 – PE (clone 205yekta), eBioscience || Thermo Fisher Scientific || Cat# 12-2051-82
Anti-mouse CD25 – PE (clone PC61) || BioLegend || Cat# 102007
Anti-mouse CD26 (DPP-4) – PE (clone H194-112) || BioLegend || Cat# 137803
Anti-mouse CD4 – PE (clone RM4-5) || BioLegend || Cat# 100511
Anti-mouse CD40 – PE (clone 1C10) || BioLegend || Cat# 12-0401-82
Anti-mouse CD44 – PE (clone IM7) || BioLegend || Cat# 103007
Anti-mouse CD62L – PE (clone MEL-14) || BioLegend || Cat# 104407
Anti-mouse CD64 (FcγRI) – PE (clone X54-5/7.1) || BioLegend || Cat# 139303
Anti-mouse CD69 – PE (clone H1.2F3) || BioLegend || Cat# 104507
Anti-mouse CD80 – PE (clone 16-10A1) || BioLegend || Cat# 104707
Anti-mouse CD83 – PE (clone Michel-19) || BioLegend || Cat# 121507
Anti-mouse CD86 – PE (clone GL1) || BioLegend || Cat# 105007
Anti-mouse CD88 (C5aR) – PE (clone 20/70) || BioLegend || Cat# 135805
Anti-mouse CD8a – PE (clone 53-6.7) || BioLegend || Cat# 100707
Anti-mouse CD9 – PE (clone MZ3) || BioLegend || Cat# 124805
Anti-mouse CLEC9A – PE (clone 7H11) || BioLegend || Cat# 143503
Anti-mouse CLEC12A – PE (clone 5d3/clec12a) || BioLegend || Cat# 143403
Anti-mouse F4/80 – PE (clone BM8) || BioLegend || Cat# 123109
Anti-mouse Ly6D – PE (clone 49-H4), eBioscience || Thermo Fisher Scientific || Cat# 12-5974-80
Anti-mouse CD301b (MGL2) – PE (clone URA-1) || BioLegend || Cat# 146803
Anti-mouse CD317 (Bst2, PDCA1) – PE (clone 927) || BioLegend || Cat# 127009
Anti-mouse PD-L1 – PE (clone MIH5), eBioscience || Thermo Fisher Scientific || Cat# 12-5982-82
Anti-mouse PD-L2 – PE (clone TY25), eBioscience || Thermo Fisher Scientific || Cat# 12-5986-82
Anti-mouse Ly-6A/E (Sca-1) – PE (clone D7) || BioLegend || Cat# 108107
Anti-mouse Siglec-F – PE (clone E50-2440) || BioLegend || Cat# 562068
Anti-mouse CD90.2 (Thy1.2) – PE (clone 53-2.1) || BioLegend || Cat# 140307
Anti-mouse Ly-6A/E (Sca-1) – BV421 (clone D7) || BioLegend || Cat# 108127

Immunocheimistry
Anti-human TCF4 – Unconjugated || Abcam || Cat# ab217668
Anti-human IRF8 – Unconjugated || Invitrogen || Cat# 39-8800
Anti-human MX1 – Unconjugated || Cell Signaling || Cat# 628155SF

IFNAR1 Blockade

InVivoMab mouse IgG1 anti-mouse IFNAR1 (clone MAR1-5A3) || BioXCell || Cat# BE0241
InVivoMab mouse IgG1 isotype control (clone MOPC-21) || BioXCell || Cat# BE0083

CITE-Seq
A2B5 || 105 || Biolegend || 150702 || AGCGAAGACGAT
CCR7 || 4B12 || Biolegend || 120101 || AGGAGTGATGAC
CCR9_CD199 || L053E8 || Biolegend || 358902 || ACCGATCTCAGC
CD103 || 2E7 || Biolegend || 121401 || CCGCGTTACACA
CD104 || 346-11A || Biolegend || 123602 || CTTAACTCATGG
CD11b || M1/70 || Biolegend || 101201 || TCAATTGCGTGC
CD11c || N418 || Biolegend || 117301 || CGTAAGAACCGT
CD127 || A7R34 || Biolegend || 135002 || CGTACAAGCCAC
CD133 || 315-2C11 || Biolegend || 141202 || CCAATACGAGCA
CD14 || Sa14-2 || Biolegend || 123302 || AAGGTGTCAGGC
CD152 || 9H10 || Biolegend || 106202 || CTGACGACTCAG
CD154 || MR1 || Biolegend || 106508 || CTCGAGTGAATC
CD16/32 || 93 || Biolegend || 101301 || CAGTTGCTCTGA
CD169 || 3D6.112 || Biolegend || 142402 || CTAGCTGACGCA
CD19 || 1D3 || Biolegend || 152402 || CATGTCTACATC
CD205 || NLDC-145 || Biolegend || 138202 || TCTGGAGGACAA
CD209a || MMD3 || Biolegend || 833001 || CAATAGCAGCTC
CD24 || M1/69 || Biolegend || 101801 || TAGTGCTAGGCG
CD25 || 3C7 || Biolegend || 101902 || AACTGCTCCACA
CD26 || H194-112 || Biolegend || 137801 || GCTTAGCTCGGA
CD273 || TY25 || Biolegend || 107202 || GACGTGGCCTAA
CD29 || HMβ1-1 || Biolegend || 102201 || TTCACTGGCTAA
CD137 || 17B5 || Biolegend || 106103 || GTCTGTAGGCAT
CD34 || HM34 || Biolegend || 128601 || GTTGAAGACTGG
CD370 || 7H11 || Biolegend || 143502 || GATCGTGTTGGC
CD38 || 90 || Biolegend || 102702 || GGCCGAGTCTAA
CD4 || RM4-5 || Biolegend || 100505 || TGACGTAACACT
CD40 || x 03/23 || Biolegend || 124601 || CAGTACGTATTC
CD44 || IM7 || Biolegend || 103001 || CTCAGATCTACC
CD45R || RA3-6B2 || Biolegend || 103263 || ACGAGGAGATGG
CD54 || YN1/1.7.4 || Biolegend || 116101 || GGACATTACCAC
CD62 || information unavailable || || || CCATGCAAGGAC
CD62L || MEL-14 || Biolegend || 104402 || AATCGCTCCGGA
CD64 || CX54-5/7.1 || Biolegend || 139301 || AAGCGTGGCTAA
CD69 || H1.2F3 || Biolegend || 104503 || ACGGCTAATCAC
CD71 || RI7217 || Biolegend || 113802 || TAGGCTGCTTAA
CD74 || In1/CD74 || Biolegend || 151002 || TATACGGACGTG
CD8 || 5H10-1 || Biolegend || 100801 || CCGATCGTATGC
CD80 || 16-10A1 || Biolegend || 104702 || AGTCATAGCCGC
CD86 || GL-1 || Biolegend || 105001 || ATGTCTAGGTAC
CD95 || SA367H8 || Biolegend || 152602 || ATCTATGCCTCC
CD97 || 18d3 || Biolegend || 105506 || AACGTAACTGAG
cKit || 2B8 || Biolegend || 105803 || CCTCGGATACTA
CLEC12A_CD371 || 5D3/CLEC12A || Biolegend || 143404 || GAACTTCTGGCG
CLEC9A_DNGR1_CD370 || 7H11 || Biolegend || 143502 || TGAGCCTCACTT
control_AH_IgG || HTK888 || Biolegend || 400901 || CATGATTGGCTC
control_mIgG1 || MG1-45 || Biolegend || 401401 || TGTCCGGCAATA
control_mIgG2a || MG2a-53 || Biolegend || 401501 || GAGGCGATTGAT
control_mIgG2b || 27-35 || Biolegend || 402201 || GATCGTAATACC
CSF1R_CD115 || AFS98 || Biolegend || 135504 || TTGATCGACCGT
CX3CR1 || SA011F11 || Biolegend || 149018 || CAATGAGTGGAA
DL4 || HMD4-1 || Biolegend || 130802 || AGGCTAAGGCAA
EpCAM || G8.8 || Biolegend || 118201 || GAGGACGATCAT
ESAM || polyclonal || R&D systems || AF2827 || ATAGGTCATGCG
F4/80 || BM8 || Biolegend || 123101 || GTCGCTTAGCAC
Flt3 || A2F10 || eBioscience || 14-1351-81 || TAGCCGATCACG
IA/IE || M5/114.15.2 || Biolegend || 107601 || TGGCTGGCTAGA
IL7Ra_CD127 || A7R34 || Biolegend || 135002 || CGGAGTAGTAAT
Jagged2 || HMJ2-1 || Biolegend || 131001 || GTGGATCATGTT
Lamp1 || 1D4B || Biolegend || 121601 || GAACTCCACCTC
Ly6A/E || D7 || Biolegend || 108101 || ATGCCAGCAGAG
Ly6C || HK1.4 || Biolegend || 128001 || AAGAGCTCGCAG
Ly6D || 49-H4 || BD Pharmingen || 557360 || CTTGTATGTAGG
Ly6G || 1A8 || Biolegend || 127601 || TCGATAACCGCT
MERTK || 2B10C42 || Biolegend || 151502 || AGCTGCCACTAC
Nectin2 || 829038 || R&D systems || MAB3869 || AACCATGGTCGC
NK1.1 || PK136 || Biolegend || 108701 || AGCAAGCCTCAT
Notch1 || HMN1-12 || Biolegend || 130602 || GCTCAGATTAGT
Notch2 || HMN2-35 || Biolegend || 130707 || CATACGCGAAGG
Notch3 || MHMN3-133 || Biolegend || 130502 || TGCTGAGGTCTT
Notch4 || MHN4-2 || Biolegend || 349002 || GCGTCCGAGAAT
PD-1 || RMP1-14 || Biolegend || 114101 || TGCTTCGCATGG

PDCA1_CD317 || 927 || Biolegend || 127002 || TTCGTACAGTTC
PDL1 || 10F.9G2 || Biolegend || 124301 || TAGGAATGCTCG
SiglecF || S17007L || Biolegend || 155502 || CGAAGAGGCCTT
SiglecH || 551 || Biolegend || 129602 || CGTGATTGAAGG
SIRPa_CD172a || P84 || Biolegend || 144003 || GAGTAGCACATA
TCRg/d || GL3 || Biolegend || 118101 || GCGACAATGACG
TNFRH3/TNFRSF26 || 509027 || R&D systems || MAB5330 || TCTCTCAAGTCC
Trop2 || polyclonal || R&D systems || AF1122 || GAACTCATAGGC
XCR1 || ZET || Biolegend || 148202 || TCTTGTAGCATG

Validation

All antibodies in the study were purchased from indicated commercial suppliers

Eukaryotic cell lines

Policy information about cell lines and Sex and Gender in Research

Cell line source(s)

The B16-FLT3L cell line used as a source of FLT3L is described and referenced in the Methods (section "in vitro DC development")

Authentication

Authenticated through the ability of its supernatant to support DC development

Mycoplasma contamination

All lines have been tested for mycoplasma and found negative

Commonly misidentified lines
(See ICLAC register)

N/A

Animals and other research organisms

Policy information about studies involving animals; ARRIVE guidelines recommended for reporting animal research, and Sex and Gender in Research

Laboratory animals

B6 CD45.1 || B6.SJL-PtprcaPepcb/BoyCrl || Charles River || #494
Cgas-/- || B6(C)-Cgastm1d(EUCOMM)Hmgu/J || Jackson Laboratory || #026554
Cx3cr1CreER || B6.129P2(Cg)-Cx3cr1tm2.1(cre/ERT2)Litt/WganJ || Jackson Laboratory || #021160
Ifnar1-/- || B6.129S2-Ifnar1tm1Agt/Mmjax || MMRRC || #032045-JAX
Mx1GFP || B6.Cg-Mx1tm1.1Agsa/J || Jackson Laboratory || #033219
OT-II || B6.Cg-Tg(TcraTcrb)425Cbn/J || Jackson Laboratory || #004194
R26LSL-EYFP || B6.Cg-Gt(ROSA)26Sortm3(CAG-EYFP)Hze/J || Jackson Laboratory || #007903
R26LSL-Tom || B6.Cg-Gt(ROSA)26Sortm14(CAG-tdTomato)Hze/J || Jackson Laboratory || #007914
Sting1-/- || C57BL/6J-Sting1gt/J || Jackson Laboratory || #017537
Tlr7-/- || B6.129S1-Tlr7tm1Flv/J || Jackson Laboratory || #008380
Tlr9-/- || C57BL/6J-Tlr9M7Btlr/ Mmjax || MMRRC || #034329-JAX
Unc93b1-/- || C57BL/6J-Unc93b13d/Mmucd || MMRRC || #010466-UCD
Ifnb1tdTom-iCre || n/a || the Stetson lab || n/a
Ifnbmob || B6.129-Ifnb1tm1Lky/J || Jackson Laboratory || #010818
Cx3cr1eGFP || B6.129P2(Cg)-Cx3cr1tm1Litt/J || Jackson Laboratory || #005582
Tcf4flox || Tcf4tm1Hmb || Dr. Dan Holmberg || n/a
Cd11c-Cre || B6.Cg-Tg(Itgax-cre)1-1Reiz/J || Jackson Laboratory || #008068

Wild animals

N/A

Reporting on sex

As stated in the Methods: Female mice were used in all experiments, except in those involving in vivo challenges (Fig. 7); these included both male and female mice similarly distributed in all treatment groups

Field-collected samples

N/A

Ethics oversight

All animal maintenance and experimentation were performed under the investigator's protocols approved by the institutional Animal Care and Use Committees of New York University Grossman School of Medicine (NYUGSoM) and University of California San Diego School of Medicine

Note that full information on the approval of the study protocol must also be provided in the manuscript.

Plants

Seed stocks	*Report on the source of all seed stocks or other plant material used. If applicable, state the seed stock centre and catalogue number. If plant specimens were collected from the field, describe the collection location, date and sampling procedures.*
Novel plant genotypes	*Describe the methods by which all novel plant genotypes were produced. This includes those generated by transgenic approaches, gene editing, chemical/radiation-based mutagenesis and hybridization. For transgenic lines, describe the transformation method, the number of independent lines analyzed and the generation upon which experiments were performed. For gene-edited lines, describe the editor used, the endogenous sequence targeted for editing, the targeting guide RNA sequence (if applicable) and how the editor was applied.*
Authentication	*Describe any authentication procedures for each seed stock used or novel genotype generated. Describe any experiments used to assess the effect of a mutation and, where applicable, how potential secondary effects (e.g. second site T-DNA insertions, mosiacism, off-target gene editing) were examined.*

Flow Cytometry

Plots

Confirm that:

☒ The axis labels state the marker and fluorochrome used (e.g. CD4-FITC).

☒ The axis scales are clearly visible. Include numbers along axes only for bottom left plot of group (a 'group' is an analysis of identical markers).

☒ All plots are contour plots with outliers or pseudocolor plots.

☒ A numerical value for number of cells or percentage (with statistics) is provided.

Methodology

Sample preparation	Described in Methods
Instrument	Described in Methods
Software	Described in Methods
Cell population abundance	Primary sorting files for each experiment are available upon request
Gating strategy	Key gating strategies shown in Fig. S6

☒ Tick this box to confirm that a figure exemplifying the gating strategy is provided in the Supplementary Information.
