# Supplementary figures and images for: Tonic type I interferon signaling optimizes the antiviral function of plasmacytoid dendritic cells
Source: Nat Immunol. 2025 Oct 14;26(11):1946–61. doi: 10.1038/s41590-025-02279-4 (PMC12662804; doi:10.1038/s41590-025-02279-4)

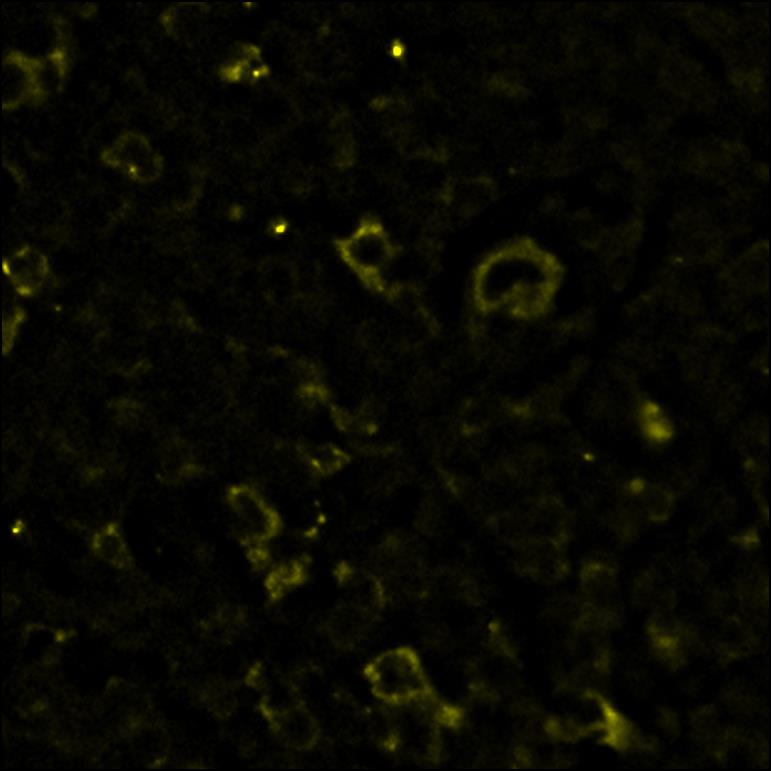

Supplement: Supplementary file 7 — Raw image files (JPEG) for Fig. 2g,h. [file 41590_2025_2279_MOESM7_ESM.zip › Pucella-Fig2-RawImages/20230710_HIPC_LN_I.3_D321_LLN/D321_LLN_MX1yellow.jpg]

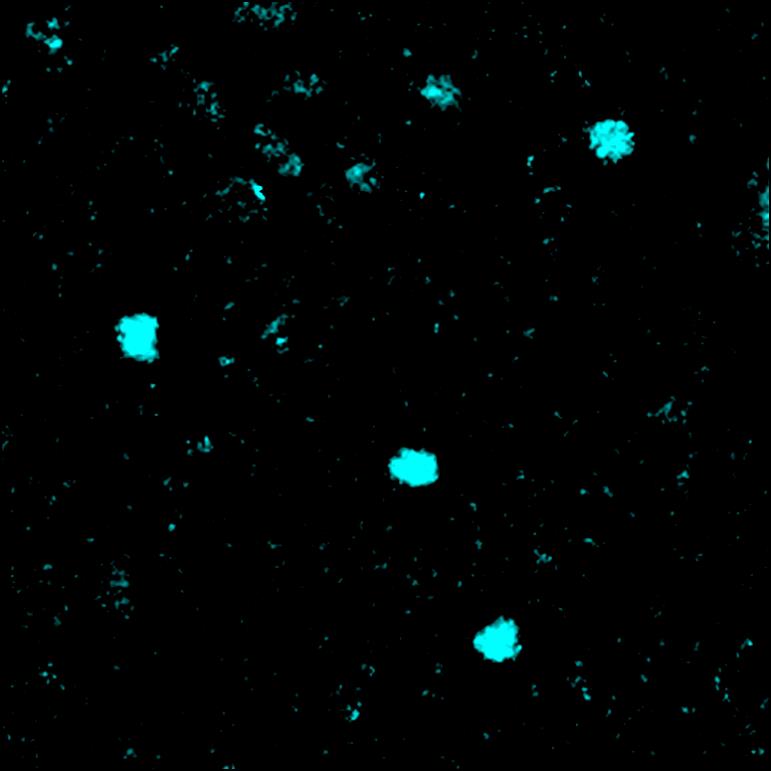

Supplement: Supplementary file 7 — Raw image files (JPEG) for Fig. 2g,h. [file 41590_2025_2279_MOESM7_ESM.zip › Pucella-Fig2-RawImages/20230710_HIPC_LN_I.3_D321_LLN/II_D321_LLN_TCF4cyan.jpg]

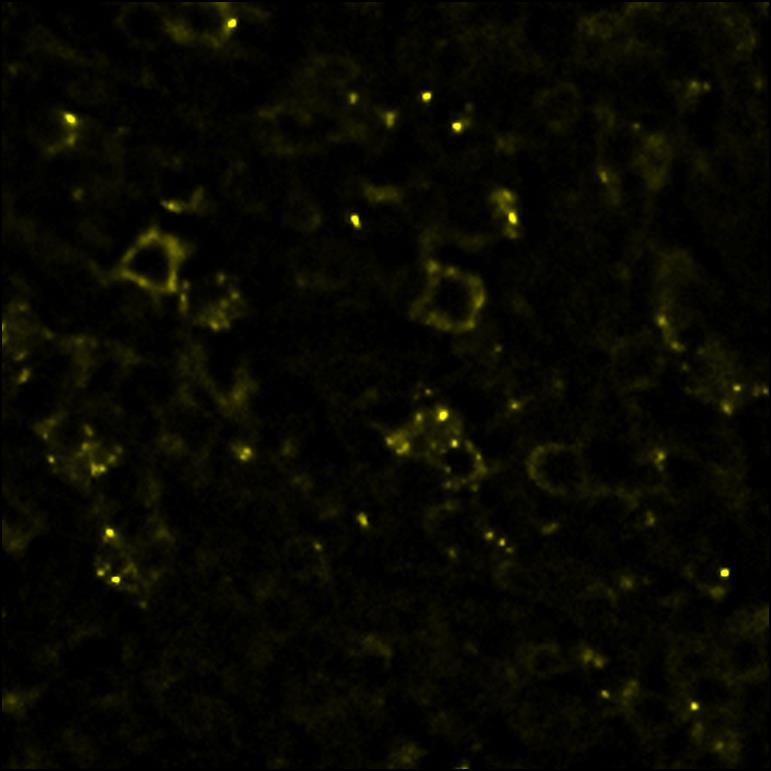

Supplement: Supplementary file 7 — Raw image files (JPEG) for Fig. 2g,h. [file 41590_2025_2279_MOESM7_ESM.zip › Pucella-Fig2-RawImages/20230710_HIPC_LN_I.3_D321_LLN/III_D321_LLN_MX1yellow.jpg]

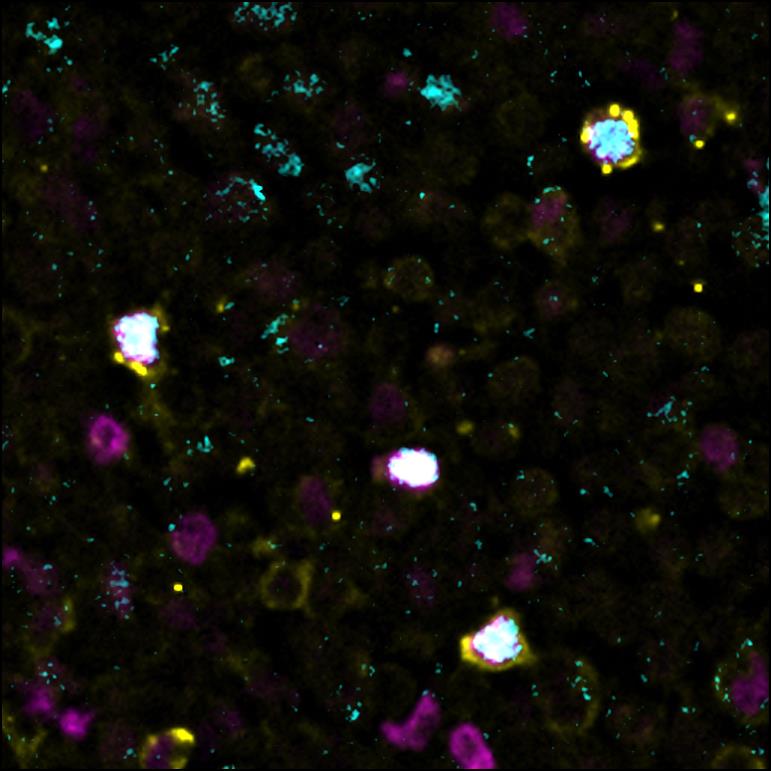

Supplement: Supplementary file 7 — Raw image files (JPEG) for Fig. 2g,h. [file 41590_2025_2279_MOESM7_ESM.zip › Pucella-Fig2-RawImages/20230710_HIPC_LN_I.3_D321_LLN/II_D321_LLN_MX1yellow_TCF4cyan_IRF8magenta.jpg]

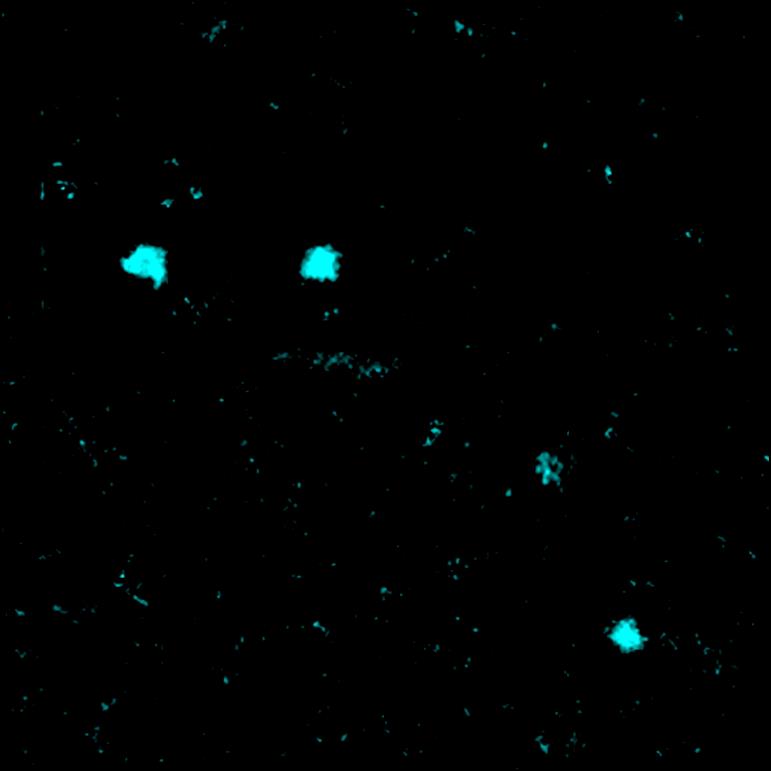

Supplement: Supplementary file 7 — Raw image files (JPEG) for Fig. 2g,h. [file 41590_2025_2279_MOESM7_ESM.zip › Pucella-Fig2-RawImages/20230710_HIPC_LN_I.3_D321_LLN/III_D321_LLN_TCF4cyan.jpg]

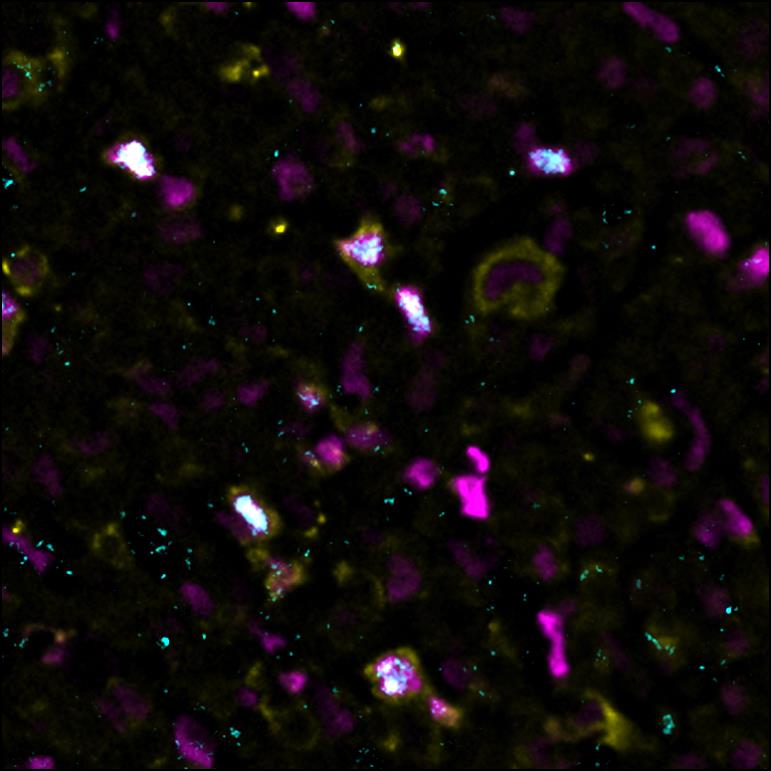

Supplement: Supplementary file 7 — Raw image files (JPEG) for Fig. 2g,h. [file 41590_2025_2279_MOESM7_ESM.zip › Pucella-Fig2-RawImages/20230710_HIPC_LN_I.3_D321_LLN/D321_LLN_MX1yellow_TCF4cyan_IRF8magenta.jpg]

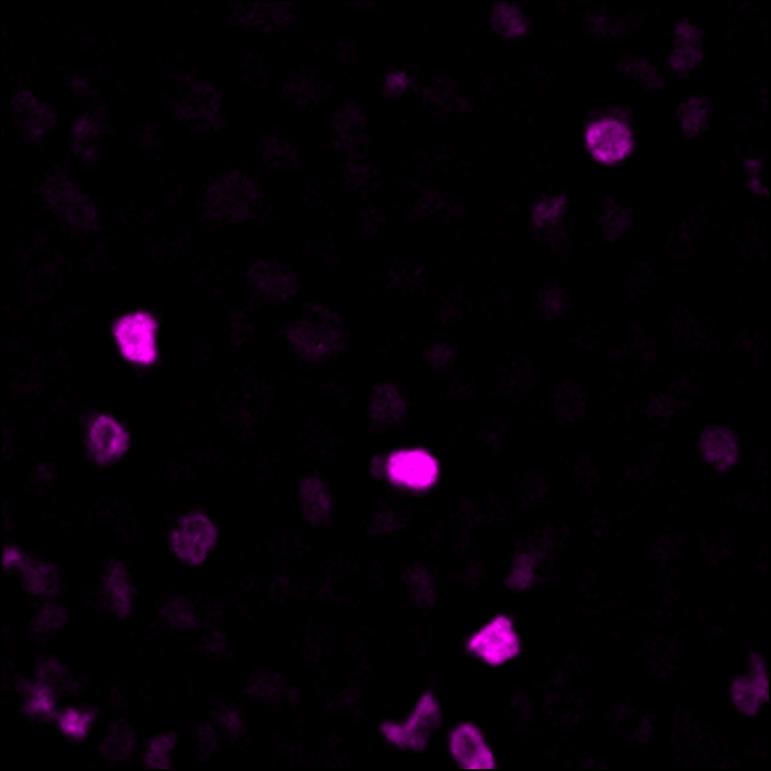

Supplement: Supplementary file 7 — Raw image files (JPEG) for Fig. 2g,h. [file 41590_2025_2279_MOESM7_ESM.zip › Pucella-Fig2-RawImages/20230710_HIPC_LN_I.3_D321_LLN/II_D321_LLN_IRF8magenta.jpg]

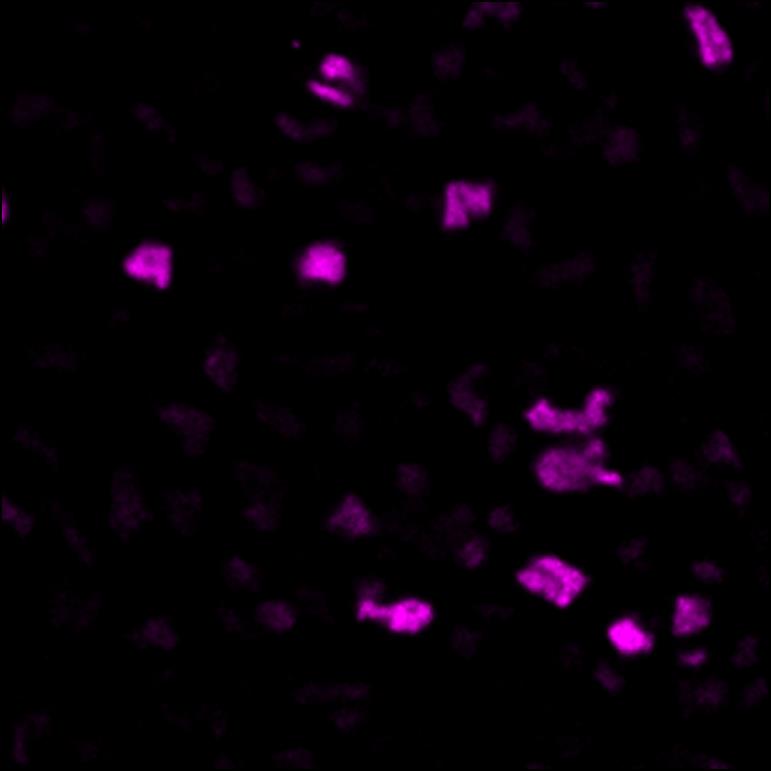

Supplement: Supplementary file 7 — Raw image files (JPEG) for Fig. 2g,h. [file 41590_2025_2279_MOESM7_ESM.zip › Pucella-Fig2-RawImages/20230710_HIPC_LN_I.3_D321_LLN/III_D321_LLN_IRF8magenta.jpg]

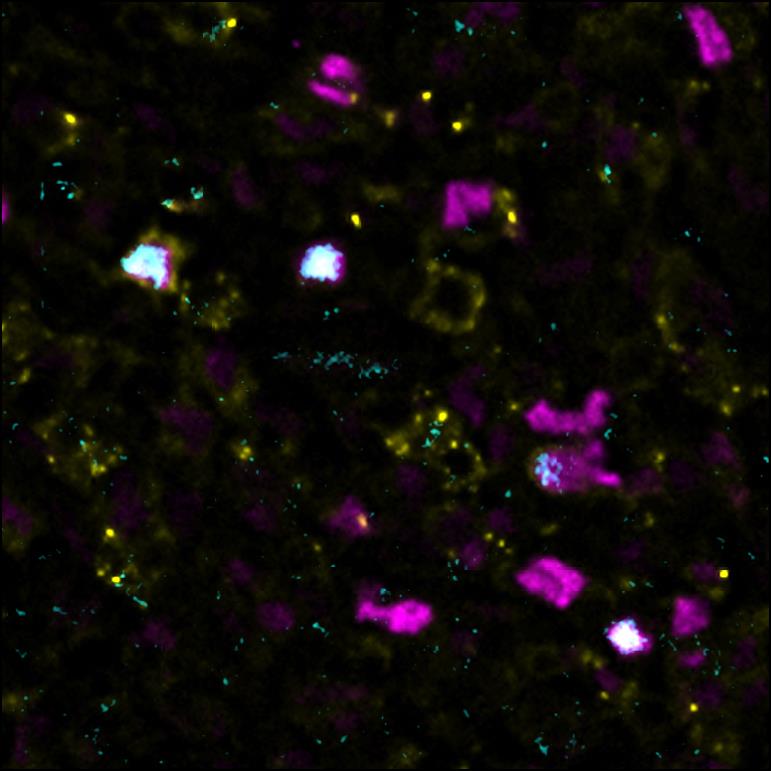

Supplement: Supplementary file 7 — Raw image files (JPEG) for Fig. 2g,h. [file 41590_2025_2279_MOESM7_ESM.zip › Pucella-Fig2-RawImages/20230710_HIPC_LN_I.3_D321_LLN/III_D321_LLN_MX1yellow_TCF4cyan_IRF8magenta.jpg]

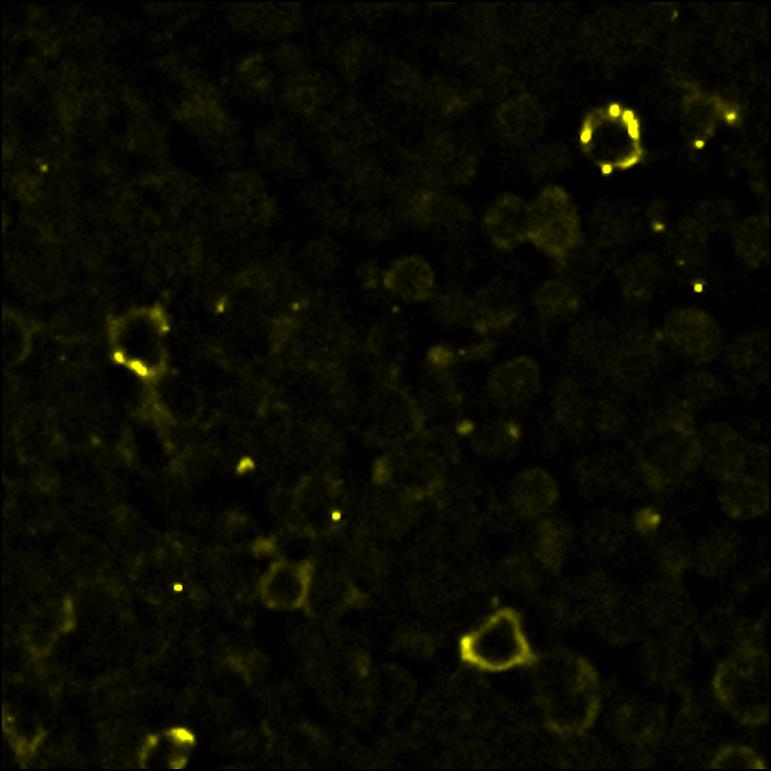

Supplement: Supplementary file 7 — Raw image files (JPEG) for Fig. 2g,h. [file 41590_2025_2279_MOESM7_ESM.zip › Pucella-Fig2-RawImages/20230710_HIPC_LN_I.3_D321_LLN/II_D321_LLN_MX1yellow.jpg]

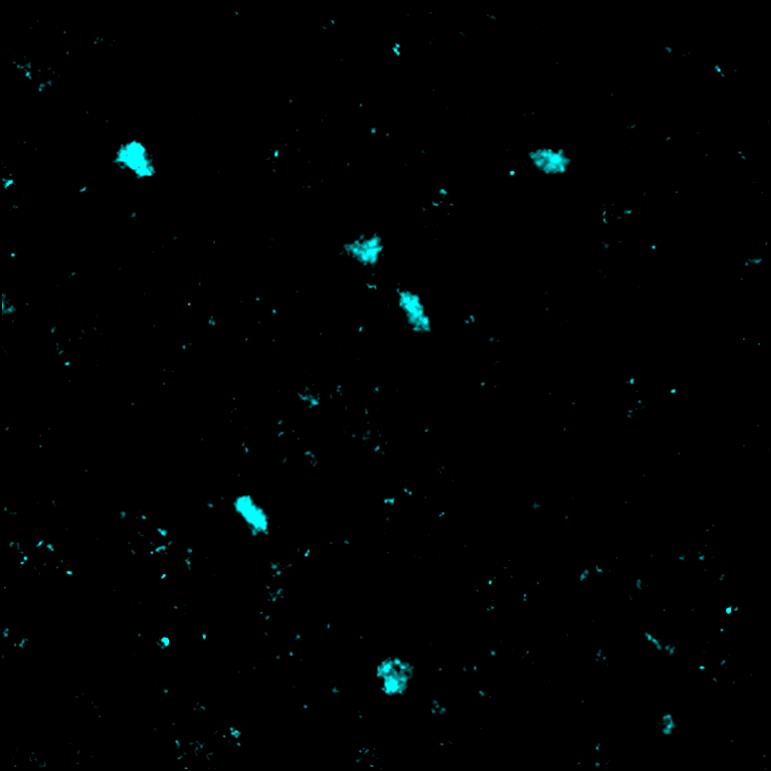

Supplement: Supplementary file 7 — Raw image files (JPEG) for Fig. 2g,h. [file 41590_2025_2279_MOESM7_ESM.zip › Pucella-Fig2-RawImages/20230710_HIPC_LN_I.3_D321_LLN/D321_LLN_TCF4cyan.jpg]

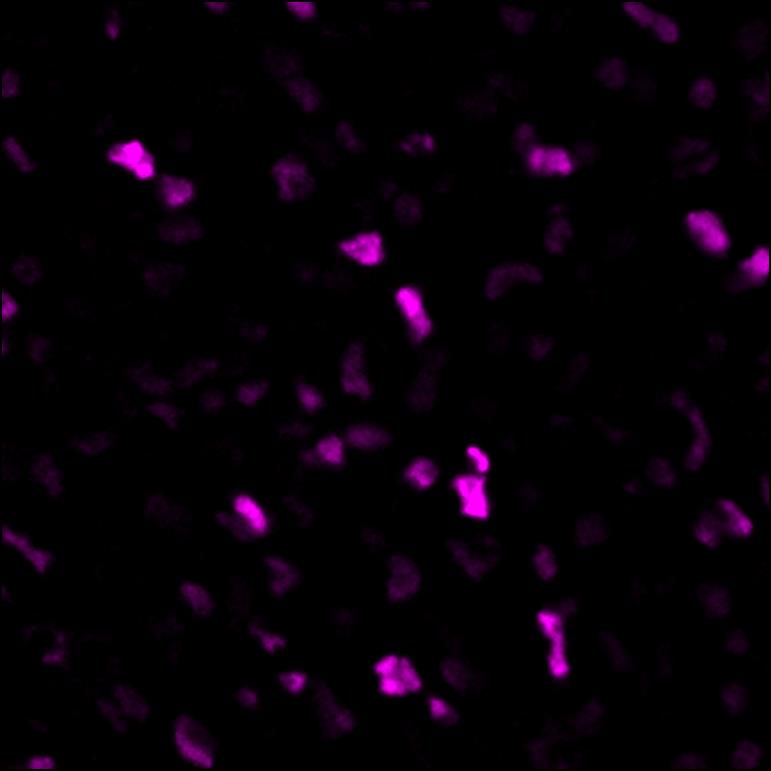

Supplement: Supplementary file 7 — Raw image files (JPEG) for Fig. 2g,h. [file 41590_2025_2279_MOESM7_ESM.zip › Pucella-Fig2-RawImages/20230710_HIPC_LN_I.3_D321_LLN/D321_LLN_IRF8magenta.jpg]

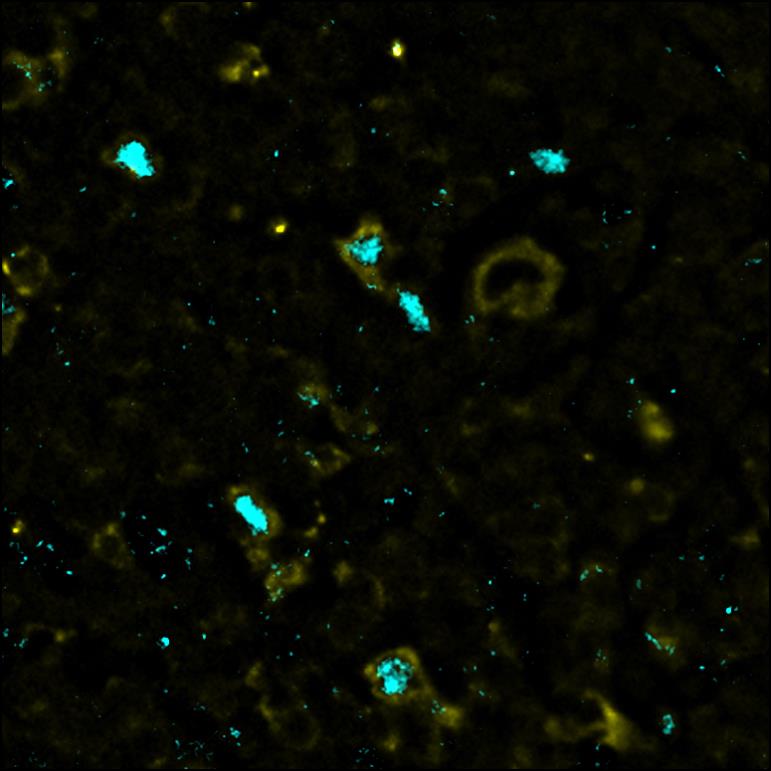

Supplement: Supplementary file 7 — Raw image files (JPEG) for Fig. 2g,h. [file 41590_2025_2279_MOESM7_ESM.zip › Pucella-Fig2-RawImages/20230710_HIPC_LN_I.3_D321_LLN/D321_LLN_MX1yellow_TCF4cyan.jpg]

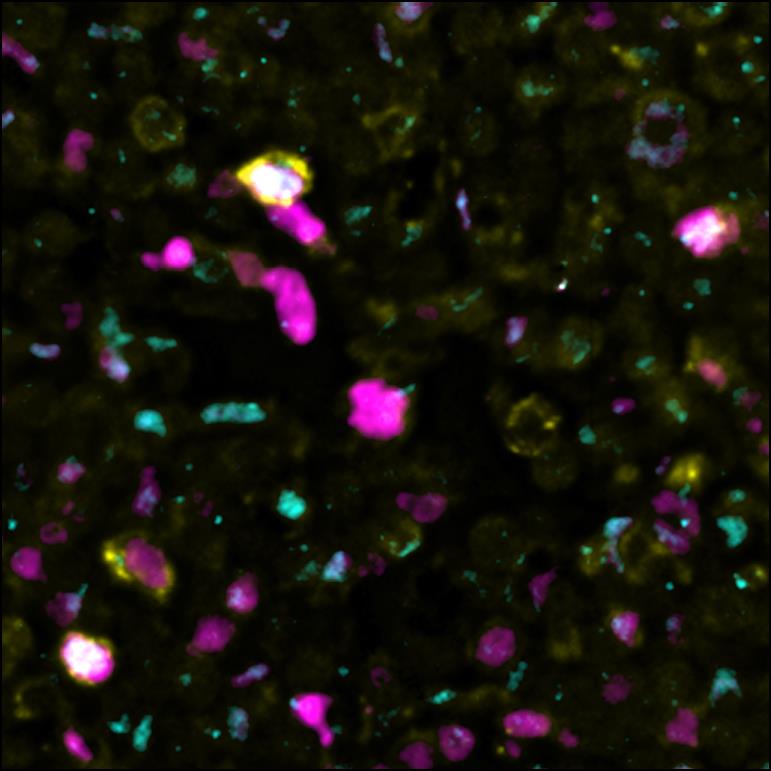

Supplement: Supplementary file 7 — Raw image files (JPEG) for Fig. 2g,h. [file 41590_2025_2279_MOESM7_ESM.zip › Pucella-Fig2-RawImages/20230507_HIPC_LN_II.5/LN_MX1yellow_TCF4cyan_IRF8magenta.jpg]

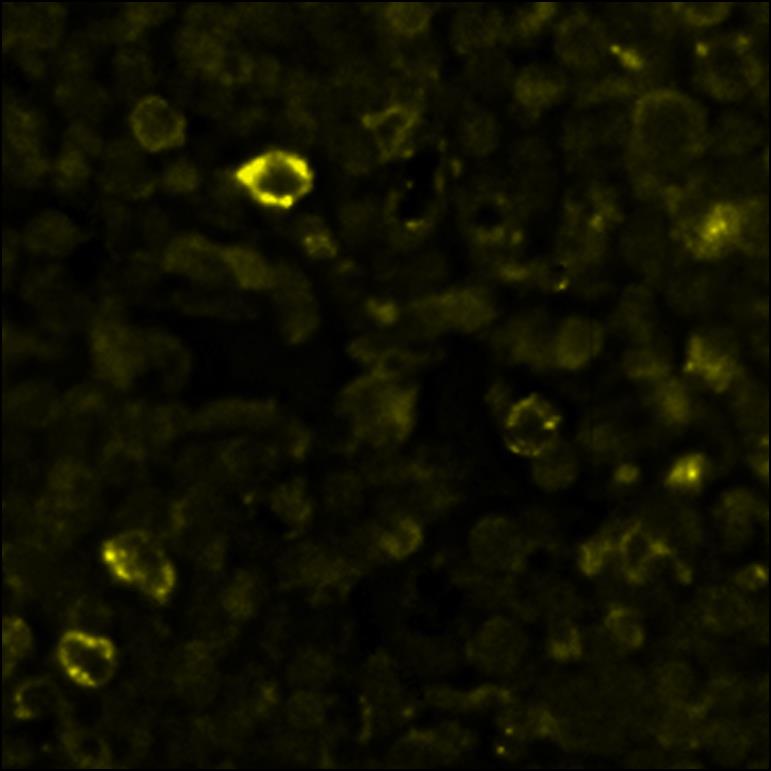

Supplement: Supplementary file 7 — Raw image files (JPEG) for Fig. 2g,h. [file 41590_2025_2279_MOESM7_ESM.zip › Pucella-Fig2-RawImages/20230507_HIPC_LN_II.5/LN_MX1yellow.jpg]

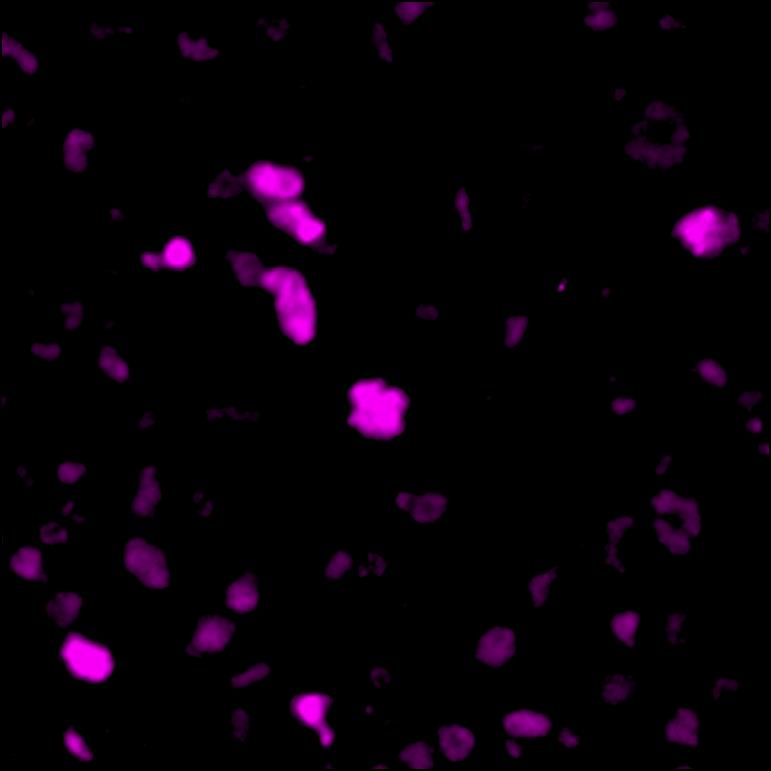

Supplement: Supplementary file 7 — Raw image files (JPEG) for Fig. 2g,h. [file 41590_2025_2279_MOESM7_ESM.zip › Pucella-Fig2-RawImages/20230507_HIPC_LN_II.5/LN_IRF8magenta.jpg]

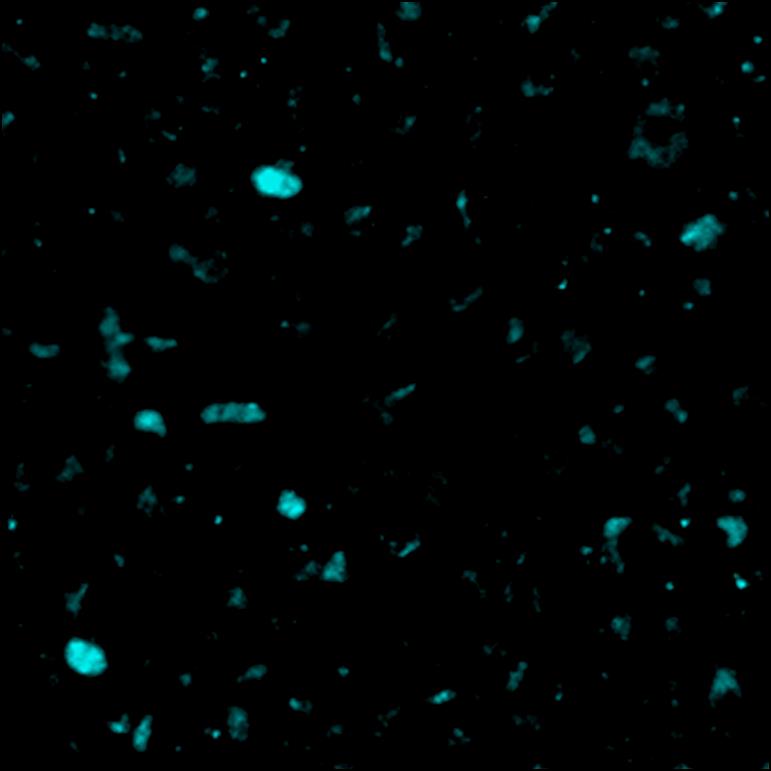

Supplement: Supplementary file 7 — Raw image files (JPEG) for Fig. 2g,h. [file 41590_2025_2279_MOESM7_ESM.zip › Pucella-Fig2-RawImages/20230507_HIPC_LN_II.5/LN_TCF4cyan.jpg]

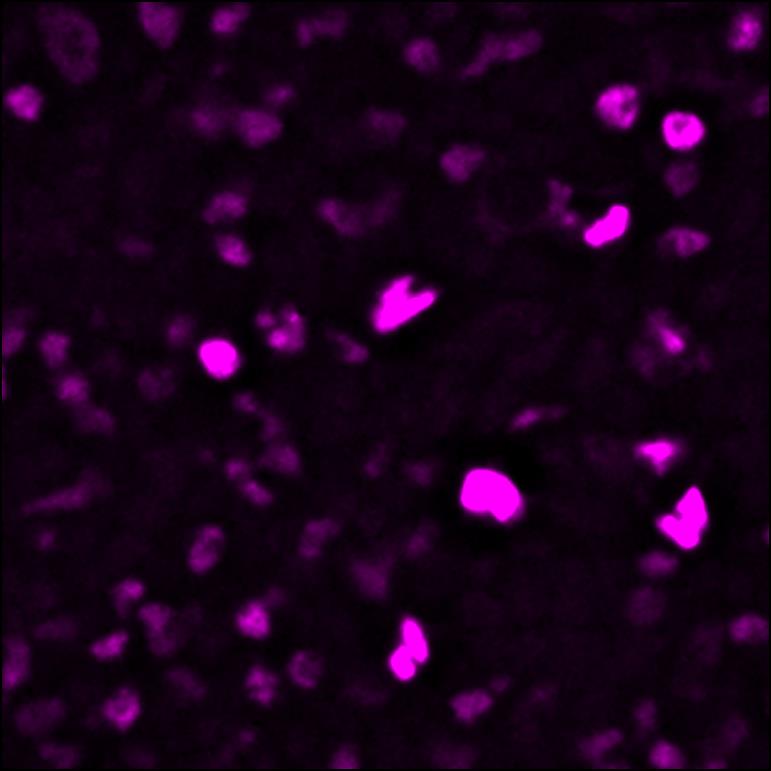

Supplement: Supplementary file 7 — Raw image files (JPEG) for Fig. 2g,h. [file 41590_2025_2279_MOESM7_ESM.zip › Pucella-Fig2-RawImages/20230714_HIPC_LN_I.6_D319_ILN/D319_ILN_IRF8magenta.jpg]

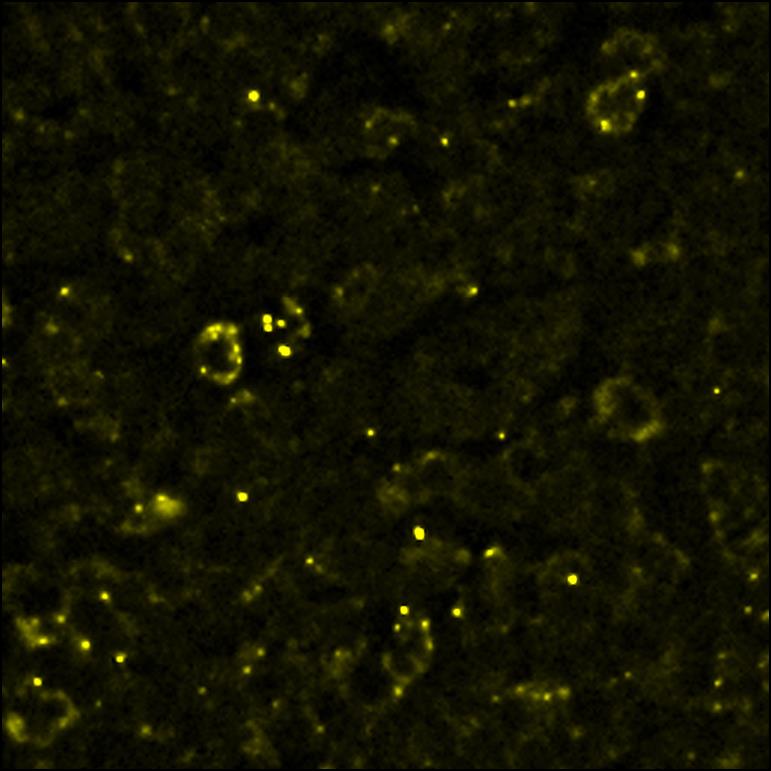

Supplement: Supplementary file 7 — Raw image files (JPEG) for Fig. 2g,h. [file 41590_2025_2279_MOESM7_ESM.zip › Pucella-Fig2-RawImages/20230714_HIPC_LN_I.6_D319_ILN/D319_ILN_MX1yellow.jpg]

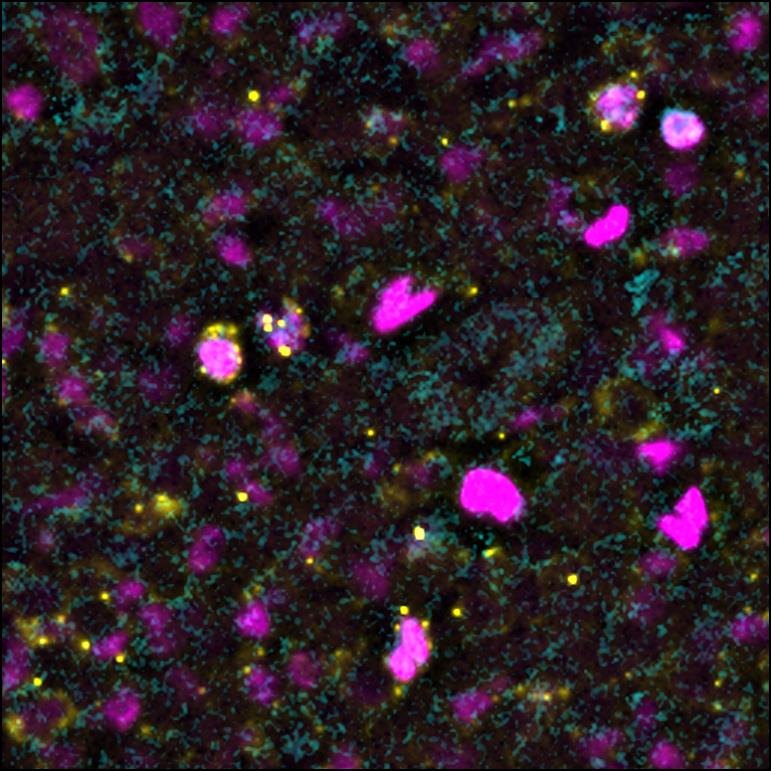

Supplement: Supplementary file 7 — Raw image files (JPEG) for Fig. 2g,h. [file 41590_2025_2279_MOESM7_ESM.zip › Pucella-Fig2-RawImages/20230714_HIPC_LN_I.6_D319_ILN/D319_ILN_MX1yellow_TCF4cyan_IRF8magenta.jpg]

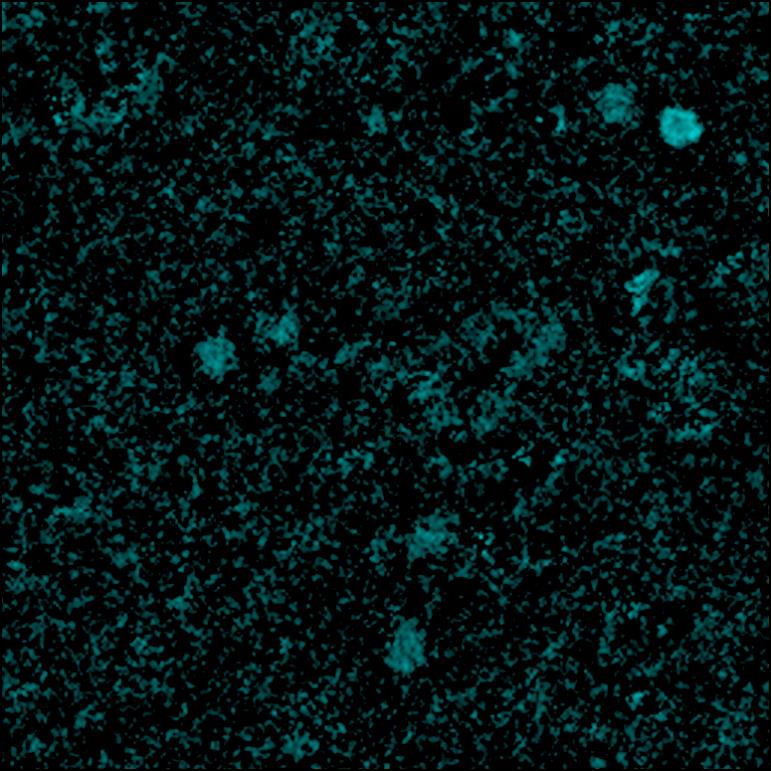

Supplement: Supplementary file 7 — Raw image files (JPEG) for Fig. 2g,h. [file 41590_2025_2279_MOESM7_ESM.zip › Pucella-Fig2-RawImages/20230714_HIPC_LN_I.6_D319_ILN/D319_ILN_TCF4cyan.jpg]

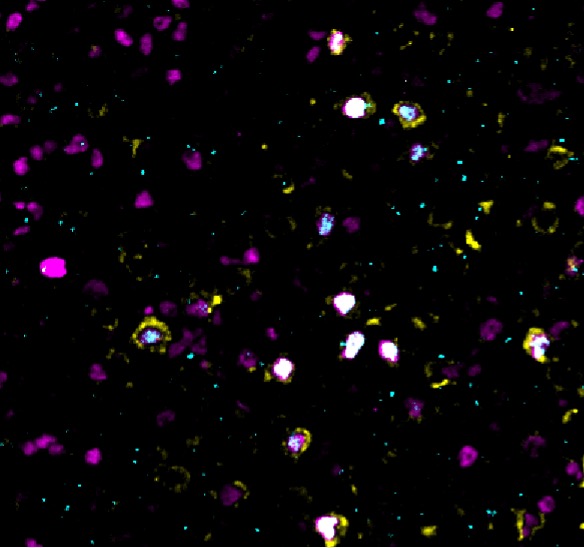

Supplement: Supplementary file 7 — Raw image files (JPEG) for Fig. 2g,h. [file 41590_2025_2279_MOESM7_ESM.zip › Pucella-Fig2-RawImages/20230814_HIPC_LN_II2_D437_PLN/20230814_HIPC_LN_II2_D437_PLN_TCF4cyanIRF8magentaMX1yellow.jpg]

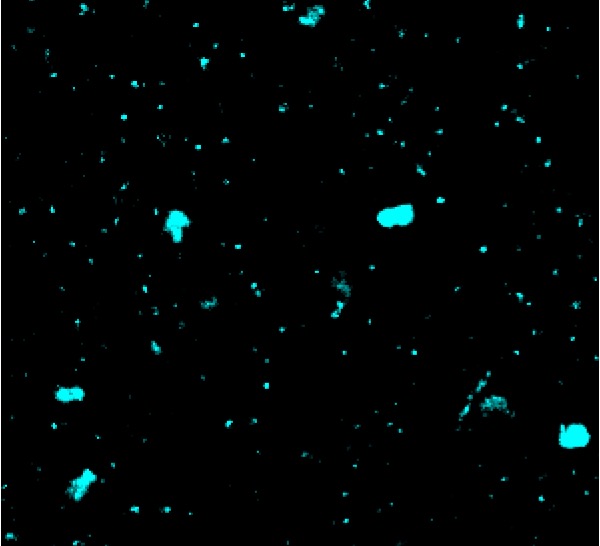

Supplement: Supplementary file 7 — Raw image files (JPEG) for Fig. 2g,h. [file 41590_2025_2279_MOESM7_ESM.zip › Pucella-Fig2-RawImages/20230814_HIPC_LN_II2_D437_PLN/20230814_HIPC_LN_II2_D437_PLN_TCF4cyan_2.jpg]

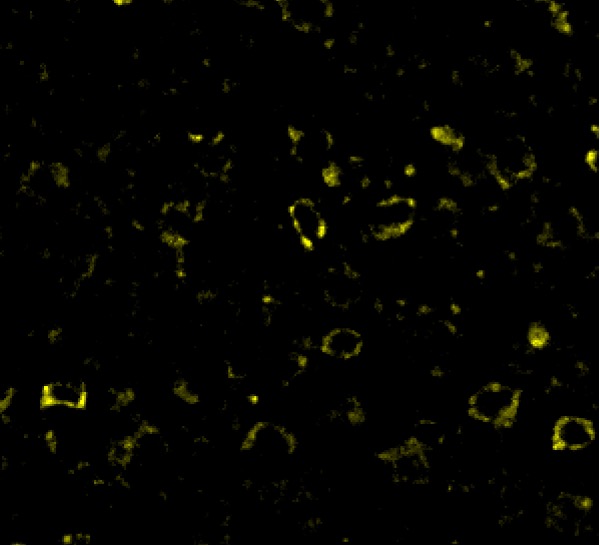

Supplement: Supplementary file 7 — Raw image files (JPEG) for Fig. 2g,h. [file 41590_2025_2279_MOESM7_ESM.zip › Pucella-Fig2-RawImages/20230814_HIPC_LN_II2_D437_PLN/20230814_HIPC_LN_II2_D437_PLN_MX1yellow_2.jpg]

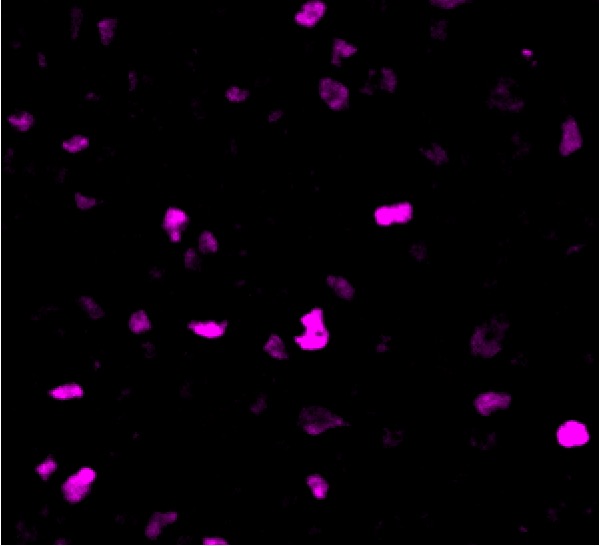

Supplement: Supplementary file 7 — Raw image files (JPEG) for Fig. 2g,h. [file 41590_2025_2279_MOESM7_ESM.zip › Pucella-Fig2-RawImages/20230814_HIPC_LN_II2_D437_PLN/20230814_HIPC_LN_II2_D437_PLN_IRF8magenta_2.jpg]

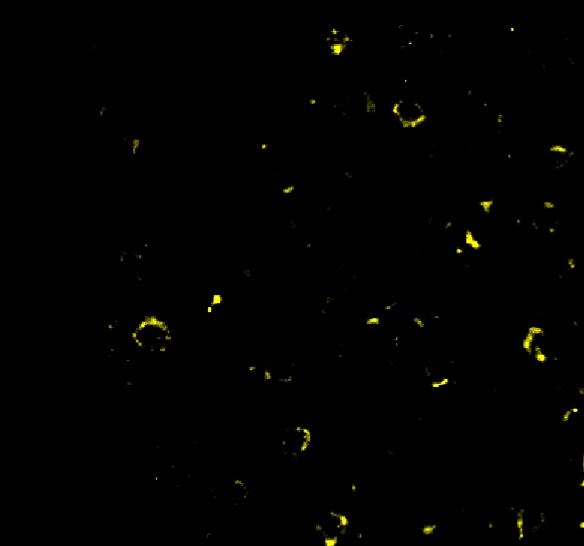

Supplement: Supplementary file 7 — Raw image files (JPEG) for Fig. 2g,h. [file 41590_2025_2279_MOESM7_ESM.zip › Pucella-Fig2-RawImages/20230814_HIPC_LN_II2_D437_PLN/20230814_HIPC_LN_II2_D437_PLN_MX1yellow.jpg]

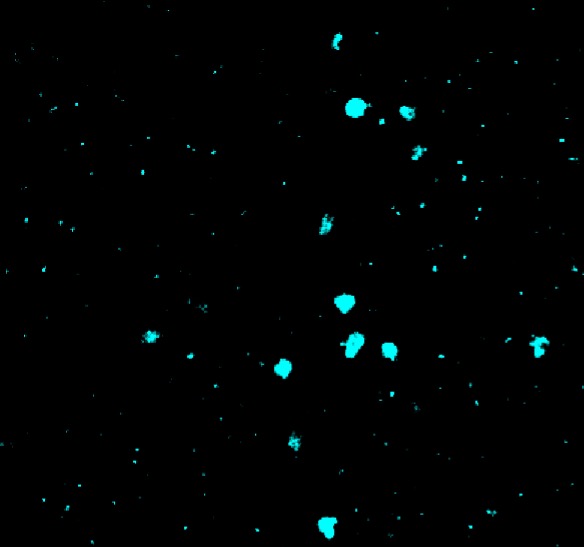

Supplement: Supplementary file 7 — Raw image files (JPEG) for Fig. 2g,h. [file 41590_2025_2279_MOESM7_ESM.zip › Pucella-Fig2-RawImages/20230814_HIPC_LN_II2_D437_PLN/20230814_HIPC_LN_II2_D437_PLN_TCF4cyan.jpg]

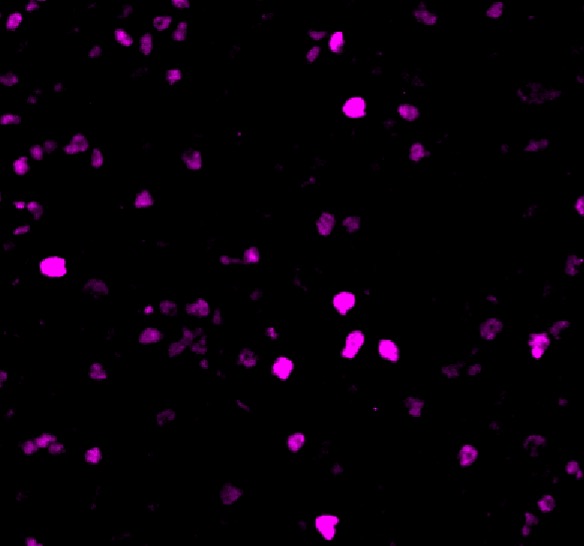

Supplement: Supplementary file 7 — Raw image files (JPEG) for Fig. 2g,h. [file 41590_2025_2279_MOESM7_ESM.zip › Pucella-Fig2-RawImages/20230814_HIPC_LN_II2_D437_PLN/20230814_HIPC_LN_II2_D437_PLN_IRF8magenta.jpg]

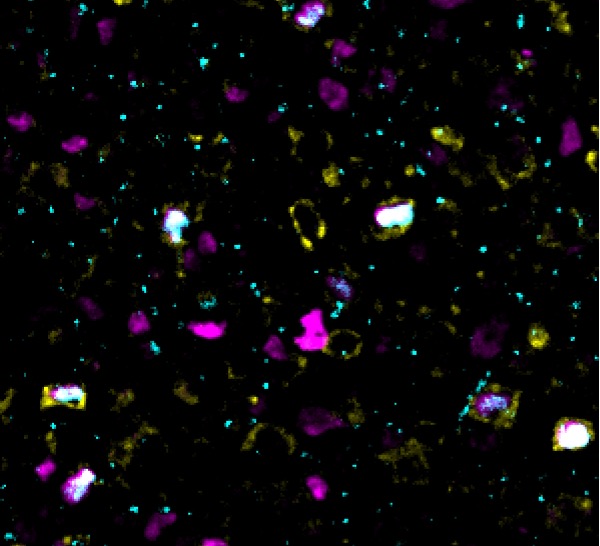

Supplement: Supplementary file 7 — Raw image files (JPEG) for Fig. 2g,h. [file 41590_2025_2279_MOESM7_ESM.zip › Pucella-Fig2-RawImages/20230814_HIPC_LN_II2_D437_PLN/20230814_HIPCLN_II2_D437_PLN_TCF4cyanIRF8magentaMX1yellow_2.jpg]
